# Bibliometric Analysis of Stem Cells in Ischemic Stroke (2001-2022): Trends, Hotspots and Prospects

**DOI:** 10.7150/ijms.86591

**Published:** 2024-01-01

**Authors:** Ting Yang, Nan Jiang, Hongxia Han, Jing Shui, Miaomiao Hou, Gajendra Kumar, Hao Tian, Lijuan Song, Cungen Ma, Xinyi Li, Zhibin Ding

**Affiliations:** 1 Department of Neurology, Third Hospital of Shanxi Medical University, Shanxi Bethune Hospital, Shanxi Academy of Medical Sciences, Tongji Shanxi Hospital, Taiyuan, 030000, China.; 2 Shanxi Cardiovascular Hospital, Shanxi Medical University, Taiyuan, 030000, China.; 3 Key Laboratory of Cellular Physiology, of Ministry of Education, Shanxi Medical University, Taiyuan, 030000, China.; 4 Department of Neuroscience, City University of Hong Kong, Hong Kong, Hong Kong SAR, 999077, China.; 5 The Key Research Laboratory of Benefiting Qi for Acting Blood Circulation Method to Treat Multiple Sclerosis of State Administration of Traditional Chinese Medicine, Research Centre of Neurobiology, Shanxi University of Chinese Medicine, Jinzhong, 030600, China.; 6 Institute of Brain Science, Shanxi Key Laboratory of Inflammatory Neurodegenerative Diseases, Medical School of Shanxi Datong University, Datong, 037000, China.

**Keywords:** Ischemic stroke, Stem cells, Bibliometric analysis, Neurogenesis, Paracrine effects, Translation.

## Abstract

**Background:** Ischemic stroke is a common cerebrovascular accident with a high risk of neurological deficits. Stem cell therapy has progressively attracted the interest of scientists and clinicians due to the benefits of promoting neural regeneration and regulating the microenvironment surrounding the lesion after ischemic stroke. Our study aimed to evaluate the development trends and research hotspots in the field of stem cells and ischemic stroke.

**Materials and methods:** Publications related to stem cells and ischemic stroke were retrieved from the Web of Science from 2001 to 2022. Data analysis and mapping were performed using VOSviewer, Citespace and ImageGP.

**Results:** In total, 1932 papers were included in the analysis. Publications have steadily increased over the past 22 years. China has contributed the maximum number of publications, whereas the USA ranked first in the total number of citations and was considered the center of the international collaboration network. University of South Florida, Henry Ford Hospital, and Oakland University were the most influential institutions. Stroke, Brain Research, and Neural Regeneration Research were the most productive journals. The research in this field was primarily focused on the effects of stem cells on neurogenesis, inflammation, and angiogenesis following ischemic stroke, as well as their therapeutic potential for the disease. In addition, neural stem cells and mesenchymal stem cells are the most commonly utilized stem cells. The topics related to miRNA, extracellular vesicles, exosomes, mesenchymal stem cells, neuroinflammation, and autophagy are current research hotspots.

**Conclusion:** Our bibliometric study provides a novel perspective on the research trends in the field of stem cells and ischemic stroke. The outcome of this study may benefit scientists to identify research hotspots and development directions, thereby advancing the application of stem cell-based therapy for ischemic stroke.

## Introduction

Ischemic stroke accounts for approximately 80% of stroke incidences and is caused by decreased cerebral blood flow with a subsequent reduction of oxygen and glucose, leading to localized cerebral ischemic necrosis or softening and corresponding impairment of neurological functions 1. It is one of the leading causes of death and disability worldwide 2. Currently, tissue plasminogen activator is the only drug approved by the Food and Drug Administration (FDA, USA) to treat acute ischemic stroke. However, the narrow time windows and high risk of haemorrhage limit the benefit to patients 3. It is urgently required for the exploration of novel alternative therapies with improved efficacy to reduce mortality and restore neurological deficits following ischemic stroke. Despite the fact that ischemic stroke can induce neurogenesis, angiogenesis, axonal sprouting, and synaptic plasticity, the sporadic endogenous repair is insufficient to compensate for the neurological defect 4. Stem cell-based therapy is an emerging therapy for neurological recovery after ischemic stroke. It is postulated to modulate neuroinflammation, augment or complement endogenous repair, and reconstruct the neural circuits 5, 6.

**Figure 1:**
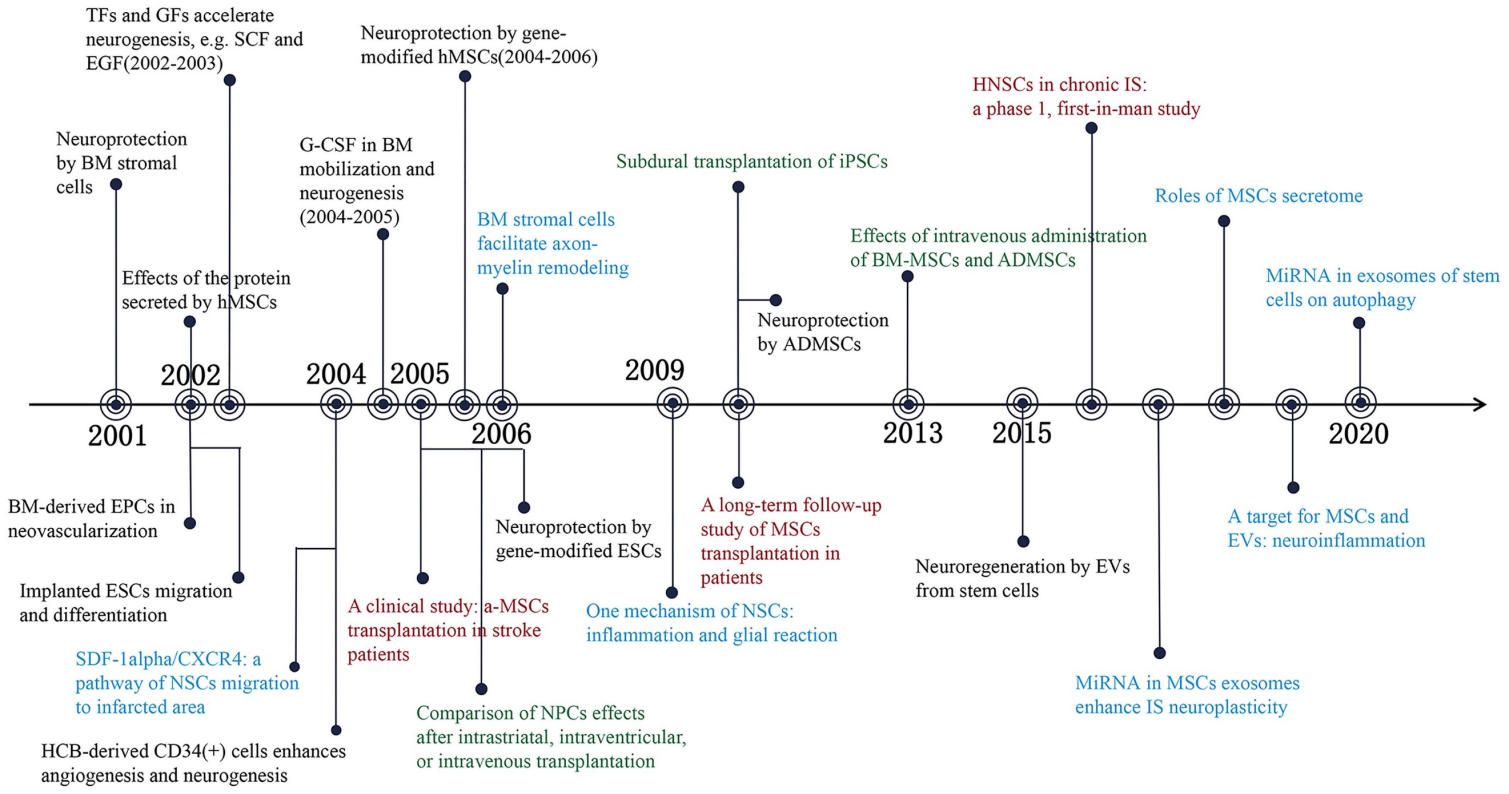
Significant findings of stem cells and ischemic stroke during 2001-2022. Events in black present observation research; Events in blue mean mechanism research; Events in red indicate clinical trials and events in green refer to transplantation routes of stem cells. BM: Bone marrow; hMSCs: Human mesenchymal stem cells; EPCs: Endothelial progenitor cells; ESCs: Embryonic stem cells; TFs: Trophic factors; GFs: Growth factors; SCF: Stem cell factor; EGF: Epidermal growth factor; SDF-1alpha: Stromal cell-derived factor 1alpha; CXCR4: CXC chemokine receptor 4; NSCs: Neural stem cells; HCB: Human cord blood; G-CSF: Granulocyte-colony stimulating factor; NPCs: Neural precursor cells; iPSCs: Inducible pluripotent stem cells; ADMSCs: Adipose derived mesenchymal stem cells; EVs: Extracellular vehicles; HNSCs: Human neural stem cells; IS: Ischemic stroke. (References were displayed on **Table S1**.)

Previous research has indicated that stem cell-based therapy is an effective method for promoting neural function recovery after ischemic stroke. Zhang et al. demonstrated that the proliferation of neural precursor cells (NPCs) in the subventricular zone (SVZ) was observed within 2 weeks after focal ischemic stroke, and that the number of NPCs decreased substantially after 4 weeks 7. The transient increase of NPCs implies an endogenous window of neuroplasticity, which offers an opportunity to improve the prognosis of ischemic stroke. Mesenchymal stem cells (MSCs), are the most extensively distributed adult stem cells and serve as an excellent therapeutic agent for the treatment of ischemic stroke 8. The neuroprotective effect of MSCs is associated with the release of bioactive substances 9. Transfection of brain-derived neurotrophic factor and placental growth factor with MSCs substantially enhanced the neurological function of ischemic rats 10, 11. Furthermore, circulating endothelial progenitor cells (EPCs) and vascular progenitor cells (VPCs) contribute to neovascularization after stroke 12, 13, while tumorigenicity limits the clinical application of embryonic stem cells (ESCs) and induced pluripotent stem cells (iPSCs) 14, 15. With the progress of stem cell research, various stem cells have been used in the preclinical study of ischemic stroke 16, 17. In addition, 86 clinical trials involving the use of stem cells to treat ischemic stroke demonstrate the potential of stem cell-based therapy in clinical settings (*clinicaltrials.gov, accessed Sep 15, 2023*). Recently, the immunomodulatory properties and beneficial paracrine effects of stem cells in ischemic stroke have garnered considerable interest 18, 19. Increased emphasis on this field has resulted in the accelerated growth of the number of publications. We summarized the influential literature related to stem cells for ischemic stroke treatment over the past 22 years, as shown in **Figure 1**. In this study, we integrated all the published literature on stem cells and ischemic stroke research, and identified research trends and hotspots in the field, which are anticipated to provide future development prospect.

Bibliometric analysis is a statistical tool that provides an overview of a specific scientific research field, describes the research trends, and predicts the prospective future directions through qualitative and quantitative approaches 20. In recent years, bibliometric analysis has been extensively used in stem cell research and the field of neuroscience 21-23. In this study, we performed a bibliometric study of the literature on stem cells and ischemic stroke published between 2001 and 2022. This bibliometric study evaluated the publication trends, countries, institutions, journals, high-cited articles, references and keywords based on basic bibliometric indicators, co-authorship networks, co-citation analysis and co-occurrence of author keywords. The mapping analysis illustrated the research trends in the field, the topics of greatest concern, and potential future research directions.

## Materials and Methods

### Data source and search strategy

**Figure 2:**
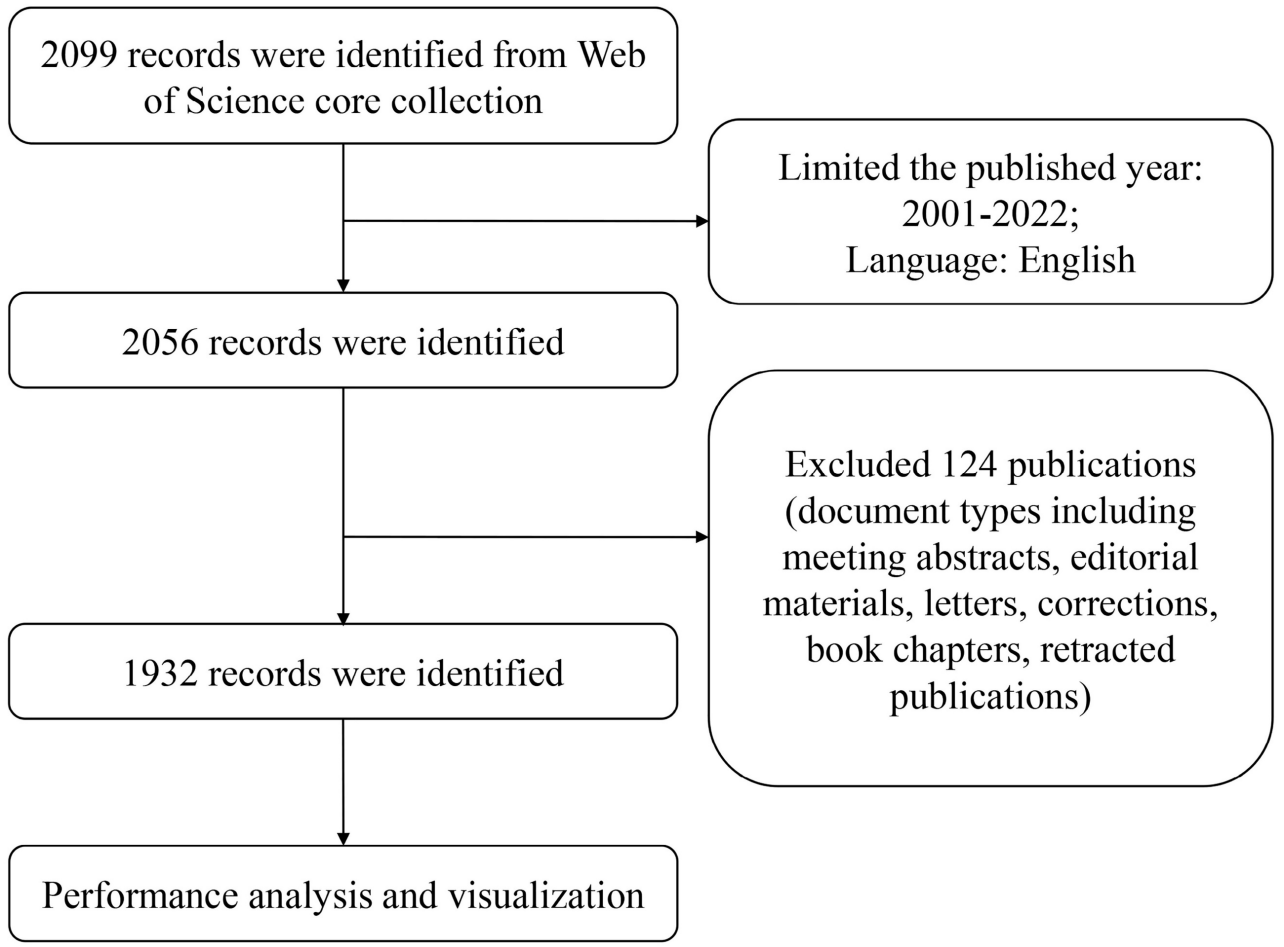
Flow diagram of publications screening.

The data was retrieved from the Web of Science (WoS) core collection, including the Science Citation Index-expanded (SCI-E) and Social Sciences Citation Index (SSCI), which is generally regarded as the most popular database for bibliometric analysis 24. The year of publications was from 2001 to 2022, and document types were limited to original articles and reviews (**Figure 2**). The searched criteria were displayed in **Table S2**. A total of 1932 published papers were retrieved and exported in the form of all records and references.

### Data standardization

Data standardization was performed on keywords and institutions in our study. The processing principles on keywords were the following: (1) Merging synonymous terms, e.g., “brain ischemia” and “cerebral ischemia”; (2) Acronyms were generally preferable, e.g., “MCAO” for “middle cerebral arterial occlusion”, similarly, “blood brain barrier” was abbreviated for “BBB”; (3) The singular and plural forms of the identical noun (e.g., Stem cell and Stem cells) were unified. The processing principles on institutions were the following: (1) Unifying the nomenclature of an institution, e.g., “Zhejiang univ affiliated hosp 2” and “Hosp 2 zhejiang univ” were unified to “Zhejiang univ hosp 2”; (2) The abbreviation was unified as the full name, e.g., “Nimh” were standardized to “National Institute of Mental Health”.

### Data analysis

Four indicators were adopted to evaluate the productivity and impact of research publications as follows: total publications (TP), total citations (TC), average citation per publication (ACPP), and H-index (which denotes that an entity has H publications, each of which has been cited a minimum of H times by other articles) 24. These indicators reflected the academic impact of a country, an institution or a journal.

VOSviewer is a well-known and optimal tool for creating and viewing bibliometric maps. In this study, VOSviewer (version 1.6.18) was used for country and institution cooperation analysis and keyword co-occurrence analysis. A measurement index clustering technique was used in the software to categorise the keywords into distinct clusters based on their association intensity and direction, and simultaneously represented each cluster by colour and time progression with varying shades 25.

CiteSpace (version 6.2.R1) was used to perform co-citation analysis of references and citation burst detection 26. The time slicing was selected from January 2001 to December 2022. Years per slice was picked by one. The selection criteria were set as follows: g-index (k=5), link retaining factor (LRF) =3.0, maximum links per node (L/N) =10, look back year (LBY) =5, e =1.0. The rest of the parameters were selected by default setting. For clustering analysis, we adapted clustering algorithms based on keywords and log-likelihood ratio (LLR) to group the literatures into conceptual clusters with different research characteristics and label each cluster with noun phrase. The colours of the clusters were determined by citing years.

ImageGP, an online data visualization tool based on R programming language 27, was employed to characterize the temporal trend of high frequency keywords in the field of stem cells and ischemic stroke.

## Results

### Timeline distribution of publications

**Figure 3:**
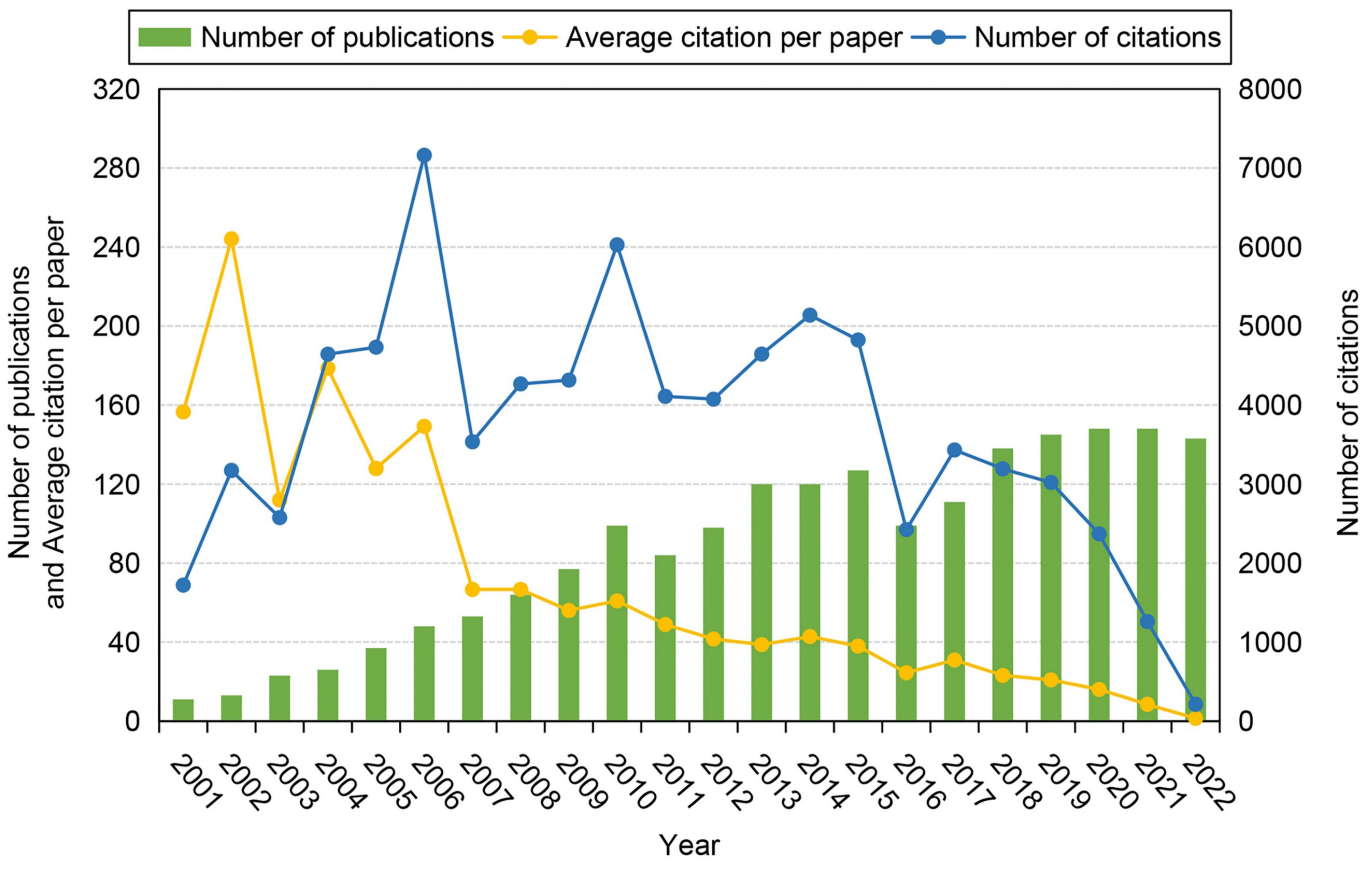
Timeline distribution of publications in the field of stem cells and ischemic stroke during 2001-2022.

**Figure 3** shows the temporal development trend in the field of stem cells and ischemic stroke over time. In the past 22 years, the number of publications has increased steadily, reaching 143 papers in 2022, which is 13 times the number published in 2001 (11 papers). This indicates that researchers have become increasingly interested in the fields of stem cells and ischemic stroke. The frequency of total citations fluctuated prior to 2005, peaked in 2006 (7162 times), and then declined progressively from 2017 to 2022. The average number of citations per paper dropped by 99.0% from 156.55 in 2001 to 1.49 in 2022.

### Analysis of countries distribution

In our database, 61 countries were identified. Countries with at least 5 publications were shown in **Figure 4A**. The main participating countries are distributed in the USA, China, South Korea, Japan and Germany. China contributed the most papers (752 papers, 38.92%) and the USA ranked first place in the total number of citations (36921) (**Table 1**). The USA had highest average number of citations per article (73.11), followed by Canada (63.75) and Germany (60.30). Overall, top 10 countries contributed 90.48% of all publications.

Subsequently, we analysed the growth trend of top 5 productive countries during the last 22 years (**Figure 4B**). USA and Japan were the most prolific countries before 2007, after which the number of publications in USA exhibited a fluctuating growth pattern whereas the growth rate in Japan was sluggish. Despite China's late entry into in this field, the number of published articles increased swiftly. After surpassing the USA in 2013, China has maintained a consistent upward trend in the number of publications over the years. In South Korea and Germany, the number of publications has been at a low.

To explore the characteristics of cooperation among countries in this field, we illustrated a collaboration network including countries with at least 5 publications (**Figure 4C**). Among 31 countries, there are 19 from Europe, 7 from Asia Pacific, 2 from the Middle East and North Africa, 2 from North America and 1 from South America. USA is at the centre of the international cooperation network, maintains deep relations with other prominent countries in this field, and has the most connections to China (87 times). However, the majority of countries lack stable and comprehensive communication and cooperation.

### Analysis of institutions distribution

We identified the 10 most productive and influential institutions in the field of stem cells and ischemic stroke, as shown in **Table 2**. University of South Florida (45 papers) was the most prolific institution, followed by Henry Ford Hospital (34 papers) and Oakland University (33 papers). In terms of total citations, Henry Ford Hospital topped the list with 4450 citations, followed by Oakland University (4414 citations). Overall, 4 of top 10 institutions were from the USA, while China, South Korea, and Japan each had two institutions.

**Figure 5:**
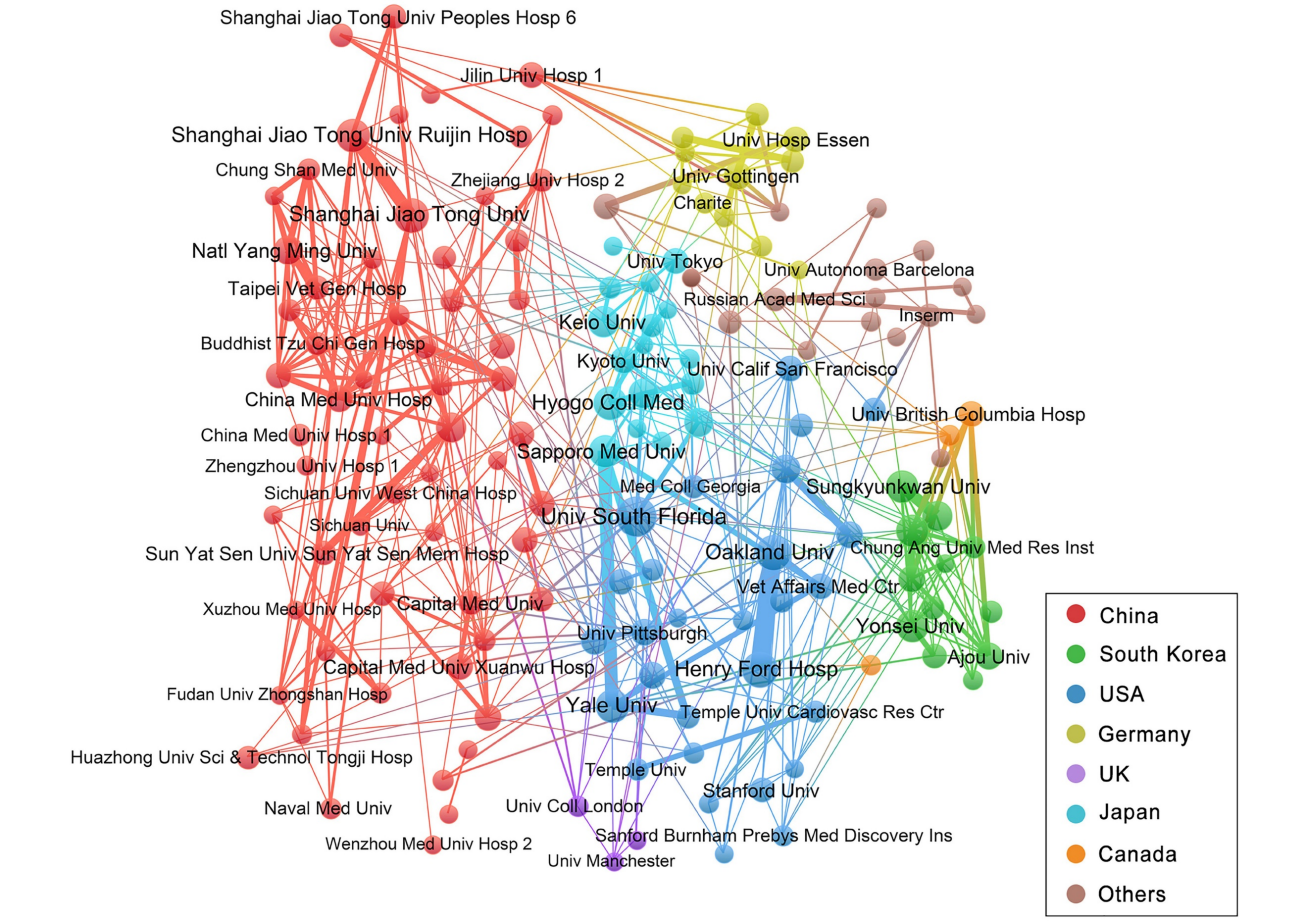
Collaboration networks of the institutions with at least 7 publications. Nodes in the network denote institutions, and the size of nodes hinges on the number of publications of institutions. The lines represent the linkages between different institutions, and the size depends on the intensity of cooperation. The colours represent different countries.

Next, we analysed the partnerships among institutions involved in this field. **Figure 5** illustrates a collaborative network that includes 151 institutions with 7 or more publications. Most of the close partnerships between institutions are from the same country, such as Oakland University and Henry Ford Hospital in the USA with 33 links, and Shanghai Jiao Tong University and Shanghai Jiao Tong University Ruijin Hospital in China with 20 links. Among transnational cooperations, Yale University, USA and Sapporo Medical University, Japan has the closest connections, and they have collaborated to publish 21 articles. In this network, 61 institutions (40.40%) come from China and 28 institutions (18.54%) from USA.

### Analysis of influential journals

537 academic journals have published articles pertaining to stem cells and ischemic stroke. The number of publications of top 10 journals in the field accounted for 24.28% (469 out of 1,932) of the total publications, as shown in **Table 3**. Stroke ranked first in all metrics, including the number of publications, total citations, citations per publication, H-index and impact factor, reflecting its preeminent position in the field. The second and third most productive journals were Brain Research and Neural Regeneration Research. Stroke and Journal of Cerebral Blood Flow and Metabolism had a total citation frequency greater than 3000. The impact factor of more than half of the top 10 journals was greater than 5. Moreover, these journals were predominantly distributed in the field of neuroscience.

### Analysis of highly cited articles

Top 10 articles with the highest citations on stem cells and ischemic stroke are shown in **Table 4**. Three articles with citation frequency above 800 were published in Journal of Cellular Biochemistry, Proceedings of the National Academy of Sciences of the United States of America, and Annals of Neurology, respectively. The article with the greatest citation frequency (2120 citations) was titled “Mesenchymal stem cells as trophic mediators” and written by Caplan in 2006. The latest publication with 557 citations was “A long-term follow-up study of intravenous autologous mesenchymal stem cell transplantation in patients with ischemic stroke”, published in Stem Cells in 2010.

### Analysis of co-cited references

**Figure 6:**
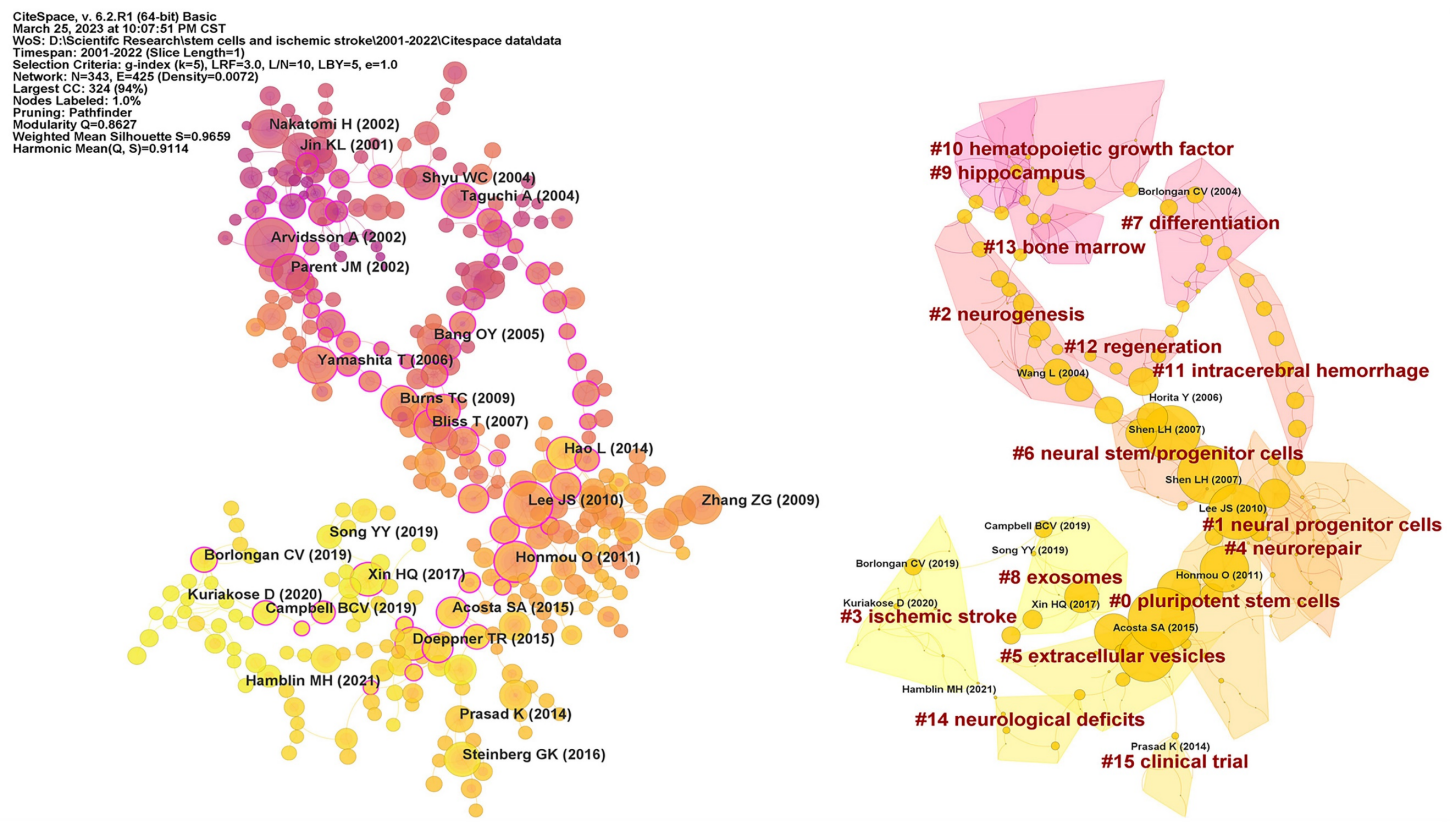
Visualization map of co-cited references and clustering network. In the left image, each circle represents a reference and the size of a circle is proportional to the citation frequency. The purple nodes reflect older references, whereas the yellow nodes represent more recent references. The betweenness centrality is denoted by a purple circle around each node. In the right image, each circle represents a reference and the size of a circle is proportional to the betweenness centrality.

The structure and dynamics of a knowledge domain are reflected in reference co-citation analysis, which also reveals the research frontiers and knowledge base 28. A total of 1182 co-cited references were identified on research of stem cells and ischemic stroke over the last 22 years. **Figure 6** displays 343 co-cited references obtained from the top 30 co-cited references per time slice for 2000-2022. The most co-cited reference was performed by Arvidsson A et al. in 2002. The article with highest centrality (0.77) was titled “Intravenous bone marrow stem cell grafts preferentially migrate to spleen and abrogate chronic inflammation in stroke” and published in Stroke in 2015. **Table 5** presented more details on top 10 references cited. All of references were co-cited at least 28 times. The most co-cited reference was an original article published in Nature Medicine, entitled “Neuronal replacement from endogenous precursors in the adult brain after stroke”.

As shown in **Figure 6**, we further recognized 15 clusters with different colours and size, which indicates that 15 different research topics have been concentrated in the field of stem cells and ischemic stroke in the past 22 years. #0 “pluripotent stem cells” and #1 “neural progenitor cells” are larger clusters. #0 includes 30 papers, primarily focusing on the efficacy and underlying mechanisms of different types of stem cells in the context of treating ischemic stroke. #1 comprises a collection of 28 papers that mostly centred around the transplantation of neural progenitor cells. These articles explored various transplantation methods, such as intravenous transplantation, intra-arterial transplantation, and intracerebral injection, among others. #10 “hematopoietic growth factor”, #9 “hippocampus”, #13 “bone marrow”, #2 “neurogenesis” were earlier research topics in this field. #8 “exosomes” and #3 “ischemic stroke” presented the latest hotspots. Among cluster emerging in recent years, the number of articles in #15 “clinical trials” was relatively small, which reflected that the correspondent research was not yet mature.

Top 20 references with the strongest citation burst were displayed in **Table 6**. Reference citation bursts emerged as early as 2002, and all references had citation burst strengths greater than 10. The reference with the strongest citation burst (strength=26.75) was titled “Neuronal replacement from endogenous neural precursors induced by ischemic stroke”, published in 2002. The reference burst spanned from 2017 to 2022 and is titled "Clinical outcomes of transplanted modified bone marrow-derived mesenchymal stem cells in stroke: a phase 1/2a Trial," highlighting one of research interests during the last 5 years. Three of the references began to burst after 2017, with the primary subject of the clinical usage of MSCs, the therapeutic potential of extracellular vehicles (EVs), and the role of miRNA in EVs, respectively. **Table S3** summarizes the main research contents of top 20 references in the order of the literature in **Table 6**.

### Analysis of keywords co-occurrence and hotspots

**Figure 7:**
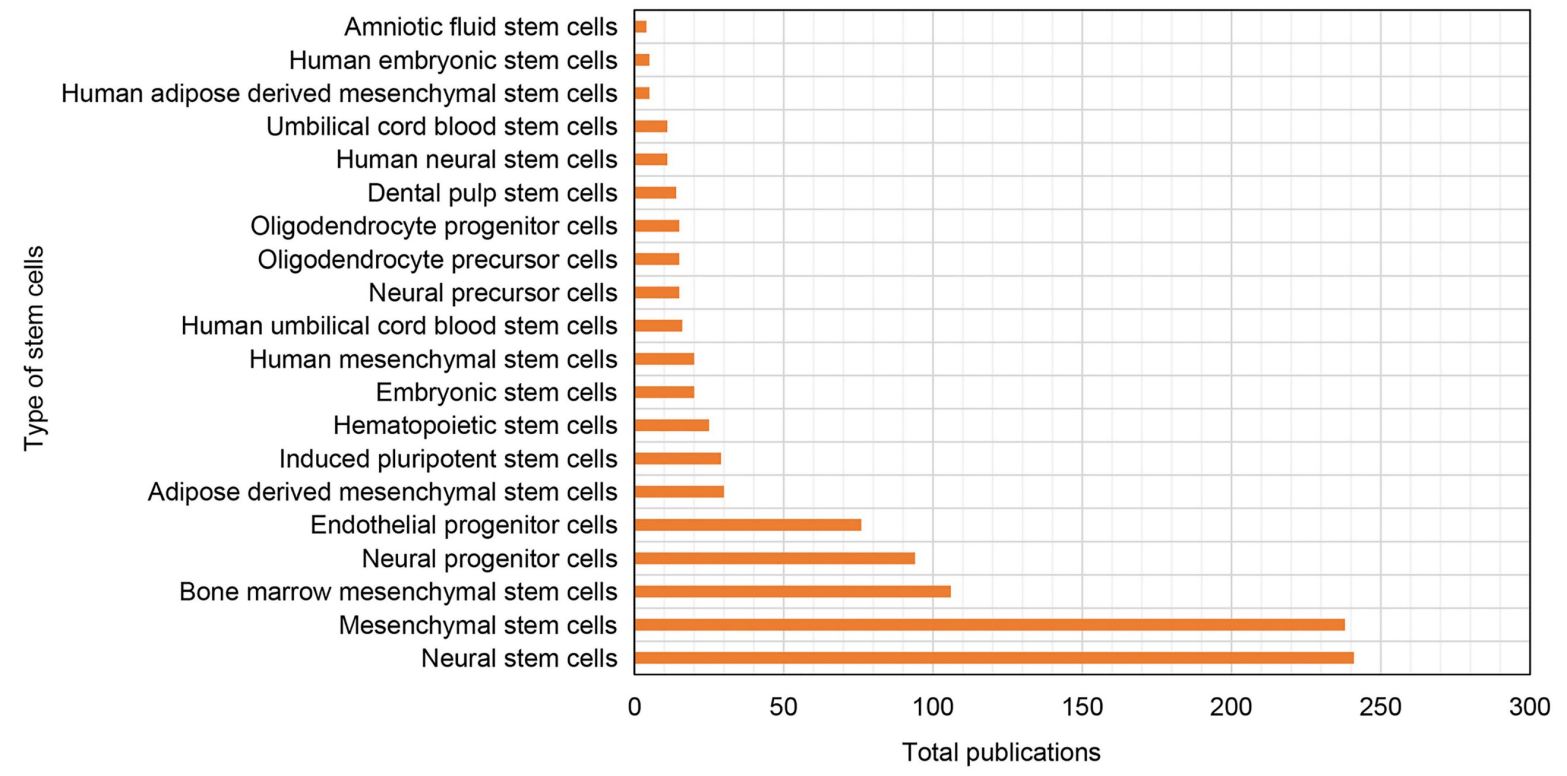
Top 20 most widely used stem cell types in the research of ischemic stroke.

A total of 1,978 keywords were identified in our dataset. We have listed 30 most frequently occurring keywords in **Table S4**. Higher occurrences of keywords such as NSCs, MSCs, neurogenesis, angiogenesis, apoptosis, and inflammation indicate that they have received considerable attention in the field. Furthermore, **Figure 7** presents the top 20 stem cell types most frequently used in research on ischemic stroke. The figure shows that NSCs and MSCs are the most commonly recognized stem cells.

**Figure 8:**
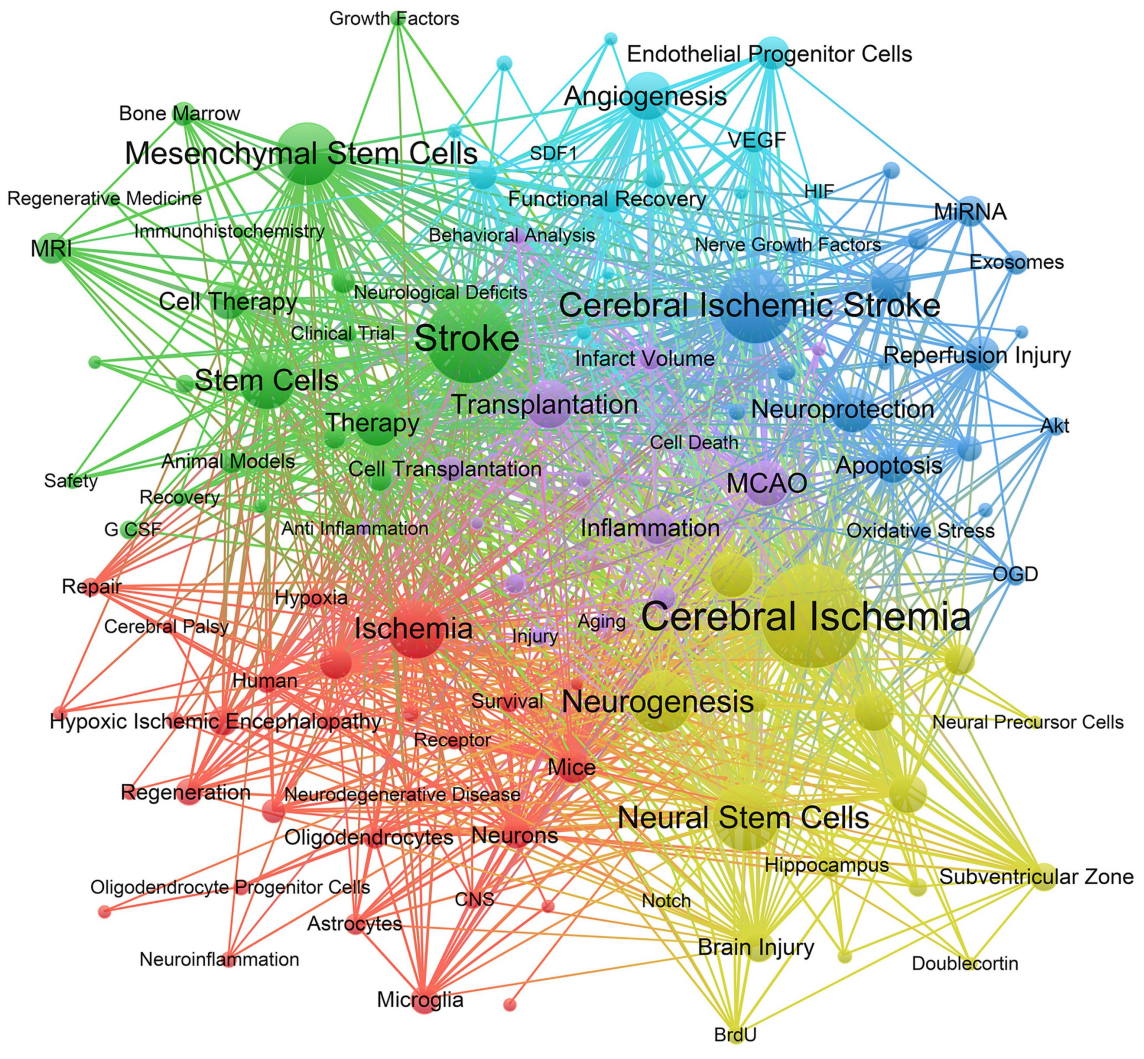
The co-occurrence network of keywords with frequencies at least 20. The size of nodes represents on the number of occurrence of keywords. The lines represent the linkages between different keywords. The colours represent different clusters.

The co-occurrence network was constructed using 116 keywords with at least 20 occurrences, as shown in **Figure 8**. The keywords in the network were divided into 6 clusters. Cluster 1 (red) focuses on the interaction between stem cells and neural cells after ischemic stroke, including keywords such as cell differentiation, neurons, astrocyte, microglia, oligodendrocytes, etc. Cluster 2 (green) shows extensive application and mechanism of MSCs in ischemic stroke, including preclinical studies and clinical trials. Cluster 3 (blue) and cluster 5 (purple) describe the effects of stem cells on inflammation and neuroprotection after ischemic stroke, with keywords such as oxidative stress, apoptosis, cell death, blood brain barrier (BBB), etc. Cluster 4 (yellow) focuses on the role of stem cells in neurogenesis following ischemic stroke, including keywords such as neural progenitor cells, nerve regeneration, subventricular zone, dentate gyrus, etc. Cluster 6 (bright blue) shows the role of stem cells in angiogenesis and the signalling molecules involved, such as the keywords endothelial progenitor cells, vascular endothelial growth factor (VEGF), etc.

**Figure 9:**
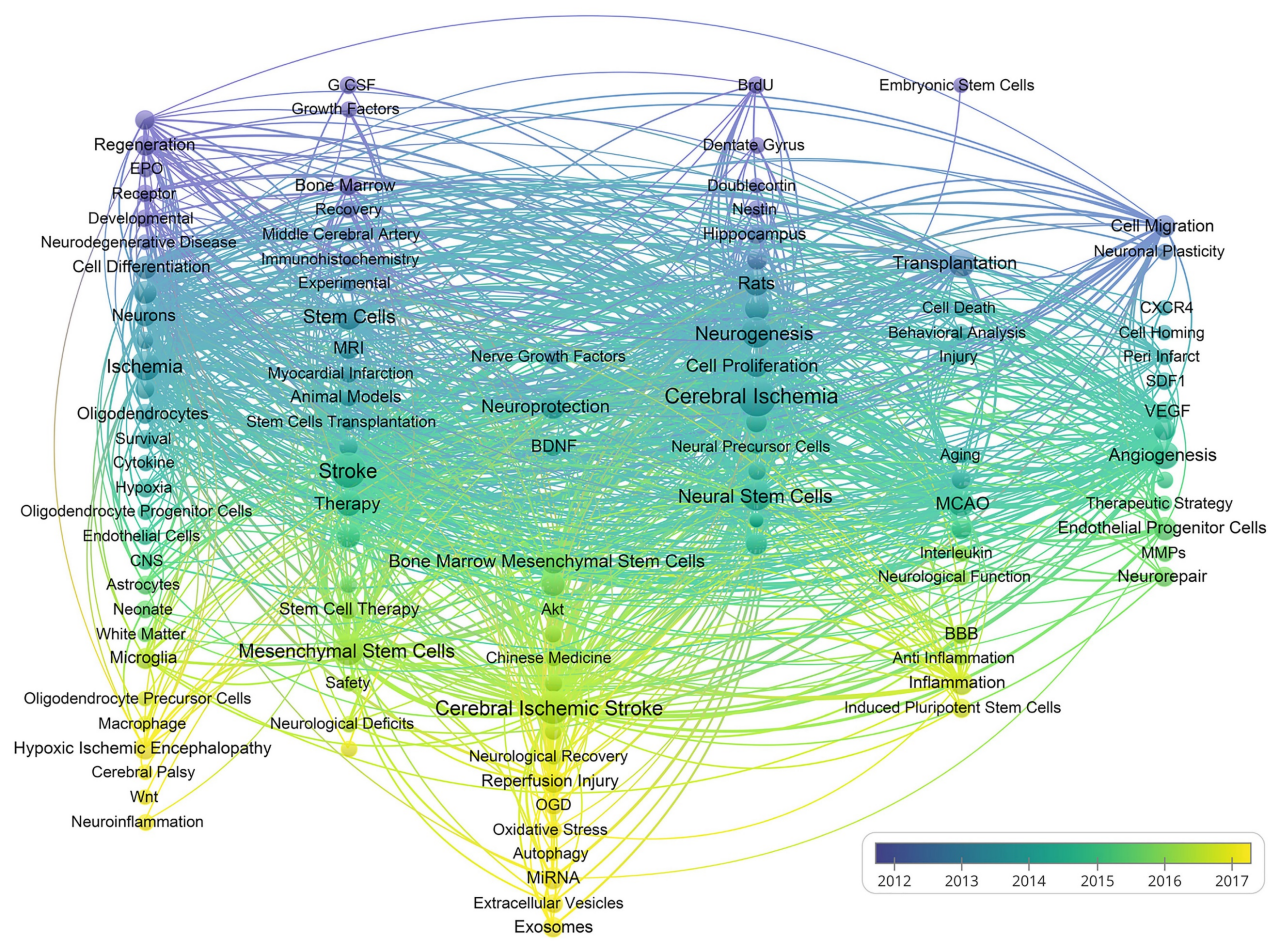
A chronological overview of the co-occurrence map of author keywords based on average appearance year.

**Figure 10:**
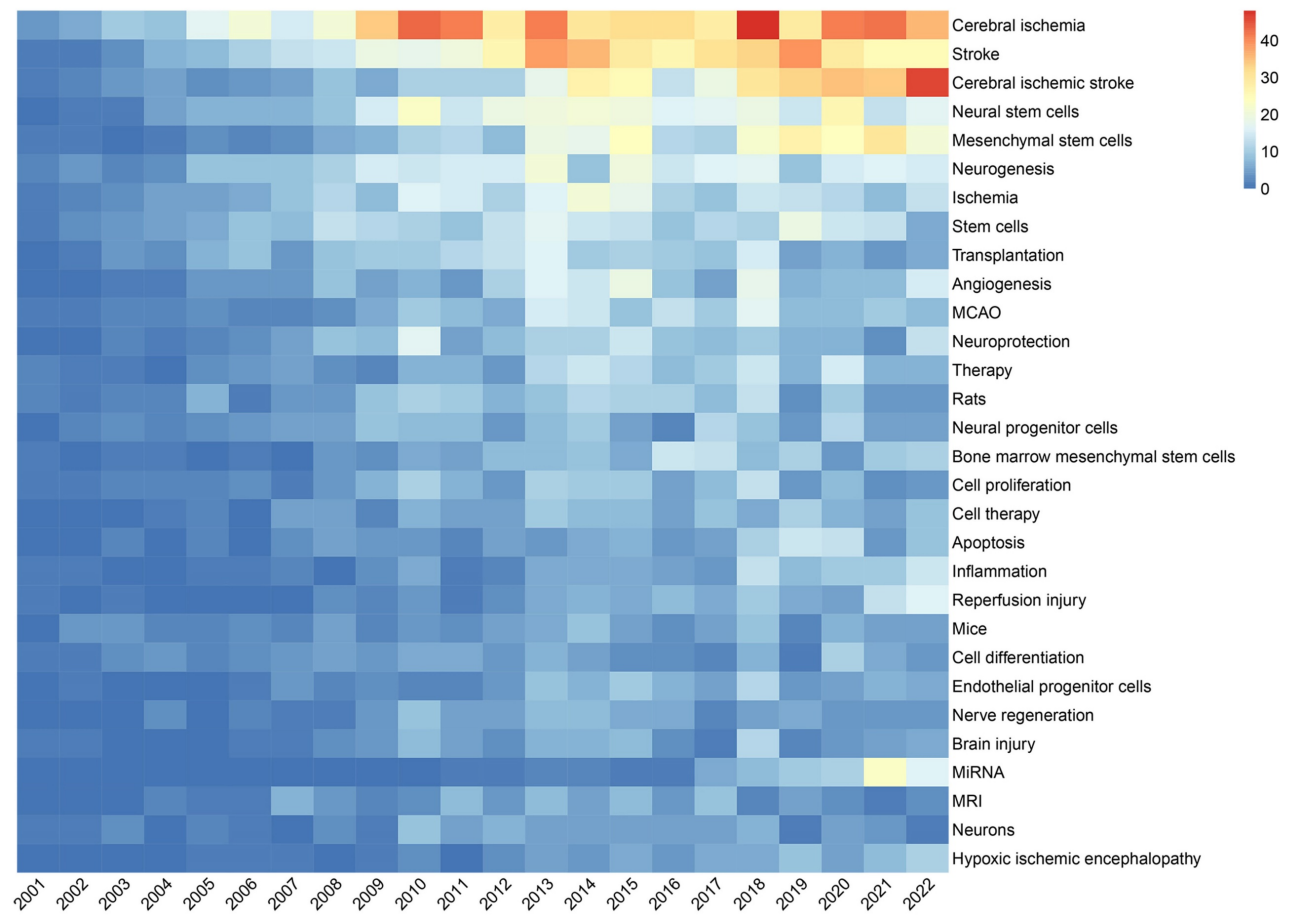
Trend of top 30 the most frequent keywords in stem cells and ischemic stroke research.

As shown in **Figure 9,** VOSviewer assigned distinct colours to keywords based on their average appearance year (AAY). In the image, blue keywords appeared before yellow ones. The keywords miRNA, EVs, exosomes, autophagy, neuroinflammation, and oxidative stress are recent hotspots. Finally, we performed a heat map analysis of time trends for the top 30 keywords (**Figure 10**). The role of MSCs in ischemic stroke has attracted the most attention in recent years.

## Discussion

### General status and scientific impact of research

In this study, we performed a bibliometric analysis of stem cells and ischemic stroke from 2001 to 2022 based on social network analysis software, presenting the research trends and progress in the field. The temporal analysis described the substantial increase in scientific output over the last 22 years. From 2001 to 2010, there was a significant increase in the number of publications, with the number of papers in 2010 being 9 times greater than that in 2001. Tremendous growth in publication suggests significant advancements in the field of stem cells and ischemic stroke in this period. However, growth rate was decelerated after 2010 and the number of publications remained steady, suggesting that the topic continues to receive great attention.

The combined publications of China and the USA constitute 60.56% of the total, which indicates a great contribution to this field by these two countries. In particular, China has published 752 papers (38.92% of total publication). It attributes to the significance of stem cell and ischemic stroke research within USA and China. In co-authorship analysis of countries, USA showed extensive cooperation with various countries, including China, Japan, and the UK. While China exhibited a higher degree of collaboration with USA as compared to other countries. This observation revealed that researchers in the United States put greater emphasis on transnational collaborative endeavors.

In institutional analysis, top 10 institutions were exclusively from top 4 countries with highest publishing output, indicating a strong correlation between a country's academic influence and achievements of its research institutions. 4 out of top 5 institutions were affiliated with USA, which reflects the dominant position of USA in this field. The extent of institutional cooperation is mainly concentrated on domestic affairs, with limited global collaboration observed among other nations. Nevertheless, this situation does not foster the advancement of the research domain. Hence, it is imperative for research institutes across different nations to enhance transnational communication in order to advance collectively for the progress of stem cell research in ischemic stroke.

The top 10 journals contributed nearly a quarter of all the area related publications. Stroke was the most productive journal followed by Brain Research, Neural Regeneration Research and Cell Transplantation. Our results indicate that the research in this field covers a wide range of disciplines, including neurosciences, cell biology, cell and tissue engineering, as well as clinical research disciplines such as clinical neurology. It has both basic research and clinical translation in this field and attracted significant interest, highlighting their potential for in-depth study and clinical application.

### Knowledge base

The thematic clusters in Figure 6 showed major themes underpinning the intellectual structure and their development over time in the field of stem cells and ischemic stroke. #10 “hematopoietic growth factor”, #9 “hippocampus”, #13 “bone marrow”, and #2 “neurogenesis” are earlier themes in stem cells and ischemic stroke research. The initial comprehension of stem cells by scientists primarily derived from the research of the hematopoietic stem cells 49. Subsequently, the appearance of #10 and #13 may be partially attributed to prior investigations conducted on hematopoietic stem cells in bone marrow 50. The hippocampus played an essential role in the initial stage to explore endogenous nerve regeneration in ischemic stroke. Therefore, understanding the mechanisms behind nerve regeneration following cerebral ischemia and identifying strategies to modulate functional neuronal regeneration have emerged as primary areas of early research. These findings offered a theoretical foundation for the application of both endogenous and exogenous stem cells transplantation.

The late research topics include #3 “ischemic stroke”, #5 “extracellular vesicles”, #8 “exosomes” and #15 “clinical trials”, illustrating the areas of focus in recent years. As stem cells transplantation has become progressively developed, several related side effects have emerged such as carcinogenicity, suboptimal transplantation efficiency, and vascular embolism 15, 51. In contrast, extracellular vesicles (EVs) derived from stem cells as a cell-free therapy have notable clinical advantages like latent tumorigenicity, low survival rate, and immune rejection of transplanted cells. In recent years, researchers in the field have displayed considerable interest in the therapeutic application potential of EVs.

#0 “pluripotent stem cells” and #1 “neural progenitor cells” are the largest clusters and prominent focus in the field of stem cells and ischemic stroke. #0 contains a diverse array of stem cell categories, with a specific emphasis on evaluating the efficacy and potential mechanisms underlying stem cells transplantation for ischemic stroke. Major stem cell types addressed within this cluster include bone marrow stromal cells, bone marrow MSCs, and EPCs, among others, which suggests that these particular stem cells have been extensively used in the treatment of ischemic stroke 52. Their safety, feasibility, and efficacy have been partially validated by animal experiments, thus exhibiting significant potential for clinical adoption. #1 primarily concerns evaluating the effectiveness of transplanting neural progenitor cells as a therapeutic approach for ischemic stroke. Neural stem/progenitor cells are well recognized as a vital cellular resource for the application of cell therapy for ischemic stroke. The selection of the stem cell transplantation route significantly influences the safety and efficacy of the therapeutic intervention. Several studies have been conducted to explore different transplantation routes, including vein transplantation, intra-arterial transplantation, and intracerebral injection 53, 54. By identifying the optimal transplantation pathway, it is possible to enhance nerve regeneration following ischemic stroke and ensure the viability of the donated cells.

### Research focus and frontiers

According to major research contents of references with strong citations bursts (Table 6 and Table S3), we can find modified MSCs transplantation in patients, EVs protective function and exosomes application enriched with miRNA cluster have received great attention in the last five years. In keywords occurrence analysis (Figure 8 and Figure 9), we discussed the role of stem cells in ischemic stroke and mostly centered around the following aspects.

### Stem cells in neurogenesis after ischemic stroke

Based on clustering analysis, it is evident that nerve regeneration has been a prominent and enduring topic of interest over the course of research. Currently, the efforts to induce neurogenesis after ischemic stroke primarily focused on endogenous neurogenesis stimulation and exogenous stem cell transplantation. Drugs such as P53 inhibitors, specific neurotrophic factors and peptides have been known to promote the proliferation and migration of NPCs in SVZ and enhance endogenous neurogenesis 55, 56. In addition, engineered biomaterials have been used to regulate drug release, stem cells reactivity and biochemical cues, which has significant therapeutic potential in neural regeneration 57. NSCs or NSCs derived from other stem cells compensated directly for the lack of endogenous neural regeneration. Several primary sources of NSCs have been utilized extensively in ischemic stroke research: a) NSCs extracted from fetal brains; b) NSCs derived from ESCs (ESC-NSC); and c) NSCs derived from iPSCs (iPSC-NSC) 58, 59. Despite several preclinical and clinical studies, it remains greatly challenging to maintain cell viability and regulate cell differentiation for NSC transplantation 60. Microenvironmental preconditioning, genetic modification, and the biomaterial assistance of NSCs have been applied to reverse the dilemma 61. Korshunova et al. proposed that gene editing techniques could inhibit the apoptosis of transplanted stem cells 62. Engineering NSCs to promote post-stroke rehabilitation bears promise for future treatment options 63.

### Stem cells in inflammation after ischemic stroke

As shown in Table 4, Imitola et al. demonstrated that inflammation guides the behavior of NSCs and recruits these cells to improve neurological deficits after brain ischemia 64. The article has been cited 856 times, suggesting it as the second most frequently cited paper among top 10 publications. It indicates that inflammatory responses have attracted significant attention of researchers in this field. In keyword clustering analysis (Figure 8), it was observed that "inflammation" was prominent keyword inside cluster 5 (purple). Additionally, cluster 3 (blue) includes the terms "oxidative stress," "apoptosis," and "cell death," which exhibit a strong correlation with inflammatory response process. Previous studies supported strong association between survival of transplanted stem cells and collective effects of reactive oxygen species and inflammatory response after stroke 65. Hence, it can be inferred that the impact of inflammation-related mechanisms plays a significant role to determine the efficacy of stem cell transplantation as a therapeutic intervention for ischemic stroke. In the frontiers and hotspots analysis (Figure 9), “anti-inflammation”, “inflammation”, and “neuroflammation” are popular keywords in recent years, reflecting that inflammation has become a significant focal point in this field. Despite the immunomodulatory role of stem cells and association with paracrine functions 66, the underlying mechanisms are not fully understood and worth to explore further.

### Mesenchymal stem cells and ischemic stroke

In recent years, MSCs have received increased attention. Our findings suggest that 3 of top 10 co-cited references (Table 5) are related to clinical trials of MSCs. In keywords occurrences analysis (Figure 8), MSCs has a frequency of 263 times, and exhibits extensive links with other keywords, which is referred as a major focus in the field of stem cells and ischemic stroke. Furthermore, the analysis of keywords evolution trends in Figures 9 and Figures 10 depicts increased interest in recent times on MSCs. Additionally, MSCs have drawn a higher level of attention over the last 5 years as compared to NSCs. This suggests that MSCs may hold important possibilities in the application of ischemic stroke.

MSCs are present in various accessible donor tissues and possess the ability to differentiate into a diverse range of cell types, including neural cells 67, 68. This characteristic provides MSCs a comparative edge over alternative stem cell types in research. Both *in vivo* and *in vitro* experiments have demonstrated that MSCs regulate the pathological processes of ischemic stroke via multiple targets. Several studies showed that MSCs enhanced collateral circulation by upregulating proteins involved in collateral remodeling and rescued damaged neurons by mitochondrial transfer and immunomodulatory effect 69, 70. MSCs are also known to facilitate axon regeneration via modulating the functioning of astrocytes and microglia 71.

Currently, there have been a total of 86 clinical trials conducted on the application of stem cells for ischemic stroke. Out of these trials, 31 of them specifically focus on the efficacy of MSCs, accounting for over one third of the total trials (*clinicaltrials.gov, accessed Sep 15, 2023*). The findings indicated that MSCs-based therapy is safe, feasible, and efficacious to treat patients with acute, subacute, and chronic ischemic stroke 72, 73. Based on the available evidence, MSCs hold greater potential as a viable option for stem cells transplantation in the treatment of ischemic stroke.

However, the influence of MSCs' secretome on cellular functions varies significantly between MSC classes 74, 75. In the absence of validated, secure, and reproducible cell extraction procedures, comparing the clinical outcomes of diverse clinical trials is difficult. Consequently, it is challenging to draw general conclusions regarding the effect of MSCs and secretome on ischemic stroke. Studies have shown that the origin, isolation, culture and amplification methods of stem cells significantly impacted the features and efficiency of MSCs 76-78. In light of heterogeneity of MSCs and high complexity of local microenvironment, it is imperative to further explore the quality evaluation method of MSCs, which is a crucial step towards clinical translation.

### Stem cells in angiogenesis after ischemic stroke

Our study suggests that angiogenesis is well recognised and prominent subject within the area. In top 10 high-cited articles (Table 4), two of them are related to the role of stem cells in angiogenesis after ischemic stroke. Ohab et.al defined a unique neurovascular niche in peri-infarct cortex, where angiogenesis and neurogenesis exhibit interdependence and mediated by specific vascular growth factors and chemokines 79. Another study confirmed the crucial involvement of human cord blood derived CD34(+) cells in promoting the environment conducive for neovascularization of ischemic brain and enhancing neuronal regeneration 36. Therefore, the presence of a favourable milieu that promotes angiogenesis is more conducive for nerve regeneration and functional recovery after ischemic stroke. The findings align with the outcomes reported in a scholarly article based on the analysis of highly co-cited references 42 (refer to Table 5). As depicted in Figure 7, beside NSCs and MSCs, EPCs are the most commonly employed types of stem cells in ischemic stroke research. This observation indicates the important role of stem cell-induced angiogenesis on neural restoration following cerebral ischemia, while also offering substantiation for the safety and effectiveness of EPC transplantation. Moreover, as illustrated in Figure 8, the term "angiogenesis" exhibits the highest frequency of occurrence and occupies a central position inside cluster 6 (bright blue). Figure 8 also shows the strong association among "Nerve growth factor", "VEGF" and "angiogenesis", highlighting their crucial role in promoting neovascularization. The discovery of stem cells derived angiogenesis-related factors and the mechanisms of post-stroke angiogenesis will contribute to a comprehensive understanding of stem cell functions in ischemic stroke.

### Extracellular vesicles and exosomes from stem cells in ischemic stroke

In top 20 references with strongest citation bursts (Table 6), Doeppnerin et al. suggests that MSCs derived EVs improved post-stroke neuroregeneration and brain remodeling, and provided valuable evidence to support the safety and efficacy of EVs in ischemic stroke 47. Based on the temporal citation burst, it may be speculated that EVs have attracted great attention from research since 2018. A study in 2017 reported that transfecting miRNA into exosomes enhanced neuroplasticity and functional recovery following ischemic stroke 48. This article had great citation burst from 2020 to 2022, reflecting the emergence of a novel topic in the field. Additionally, it was reported that #5 extracellular vesicles and #8 exosomes are latest main focus of research (Figure 6). Furthermore, Figure 9 indicates that AAY of EVs and exosomes is the most recent, which suggest that they are emerging as prominent areas of interest within this field. Therefore, EVs and exosomes may serve as a novel target for future research to evaluate the therapeutic potential in ischemic stroke.

A series of current studies have demonstrated that stem cell-derived exosomes play various roles in promoting neural repair after ischemic stroke, including inhibiting cell apoptosis and the inflammatory response, secreting angiogenesis factor, and stimulating axon regeneration and remodeling 80, 81. As a cell-free therapy, EVs have significant clinical advantages over the latent tumorigenicity, low survival rate, and immune rejection of transplanted cells. However, there is no standard protocol for exosome collection, storage, transportation, or delivery 82. These unresolved issues can serve as prospective research attempts.

### Advantages and limitations

We performed a comprehensive bibliometric analysis of publications in the field of stem cells and ischemic stroke and observed an increasing attention in this domain. First, the distribution and collaboration network of publications depicted the most influential countries and institutions. Encouraging profound cooperation among these prominent national institutions could enhance the advancement in this field. Second, analysis of keywords provides precise and deeper understanding of unique impacts of stem cells involved in the progression of ischemic stroke. It also identified several forefront topics of interest. Additionally, NSCs and MSCs are the most common types of stem cells utilised in clinical research. Safety, feasibility, and efficacy have been evaluated by previous clinical trials of ischemic stroke patients. Therefore, it is potentially promising option to further focus on stem cells with the aim of detailed investigation to ascertain effective therapeutic strategies for ischemic stroke.

However, it is irrefutable that our study has several limitations. Firstly, WoS database was only used to search relevant publications, whereas publications in other databases, such as PubMed and Scopus, may have been overlooked in our study, potentially introducing selection bias. Secondly, due to limited literature types and languages, our search strategy may not identify all the relevant references. Moreover, as the database is continuously updated, there is a discrepancy between the search results of this study and the actual number of articles included. However, we believe that comparable studies in the future will be able to resolve these limitations to the greatest extent.

## Conclusions

Our bibliometric study depicted the global situation and research growth trends in the field of stem cells and ischemic stroke over the past 22 years. We showed that research on stem cells and ischemic stroke has received great attention in recent years, and the number of publications has increased steadily. We also identified the most influential country, institution, and journal in the field. Existing research focuses primarily on neurogenesis, inflammation, angiogenesis, EVs, MSCs and therapeutic efficiency of stem cells. However, there are still several unresolved issues with stem cell clinical translations. Topics related to miRNA, EVs, exosomes, MSCs, neuroinflammation and autophagy may represent future research frontiers. Our findings contribute to identify research hotspots and trends, which provide novel opportunities for further study and application of stem cells in ischemic stroke.

## Supplementary Material

Supplementary tables.

## Figures and Tables

**Figure 4A:**
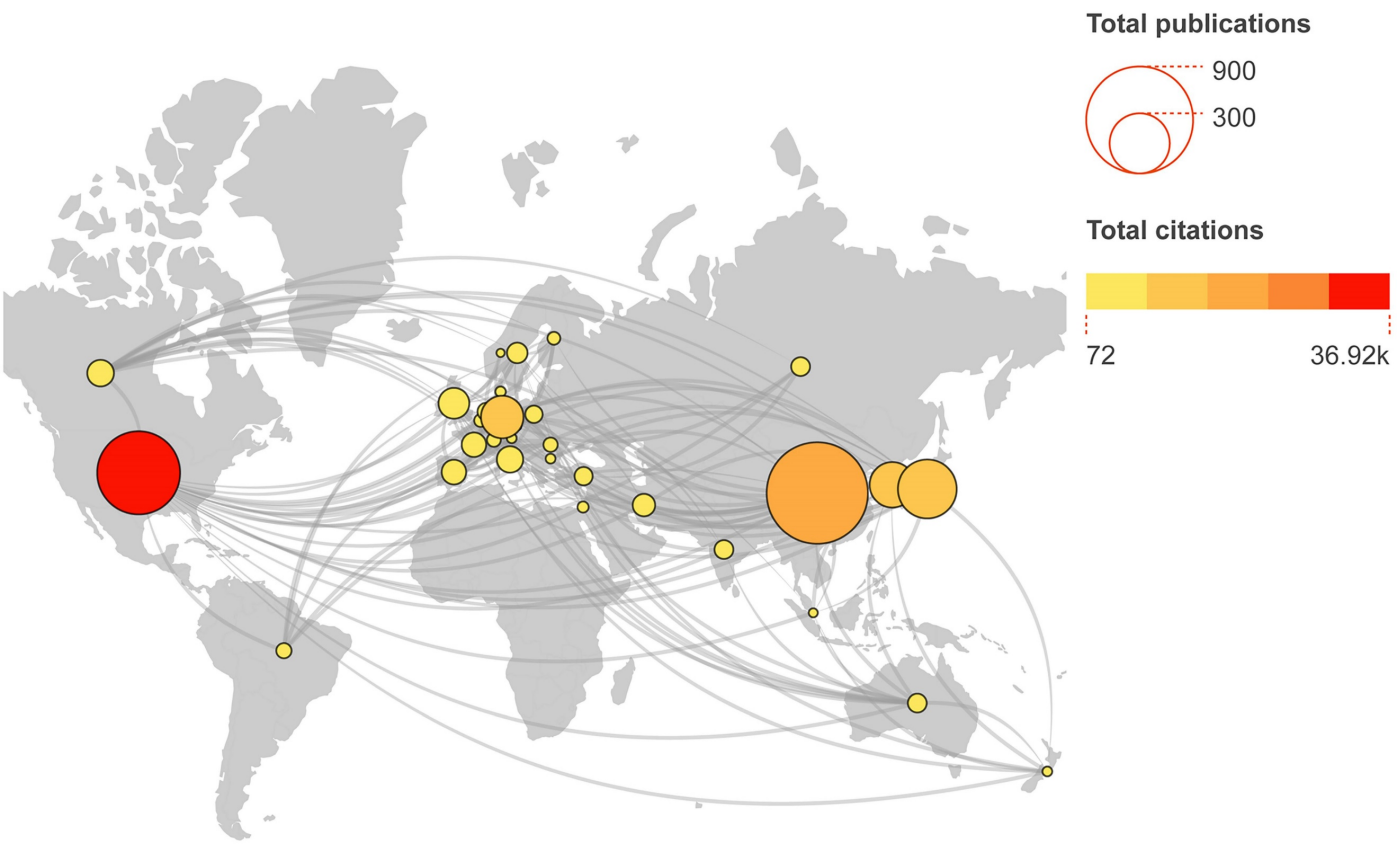
Global collaboration and distribution of publications in the field of stem cells and ischemic stroke. The number of publications and total citations are represented, accordingly, by node sizes and colours.

**Figure 4B:**
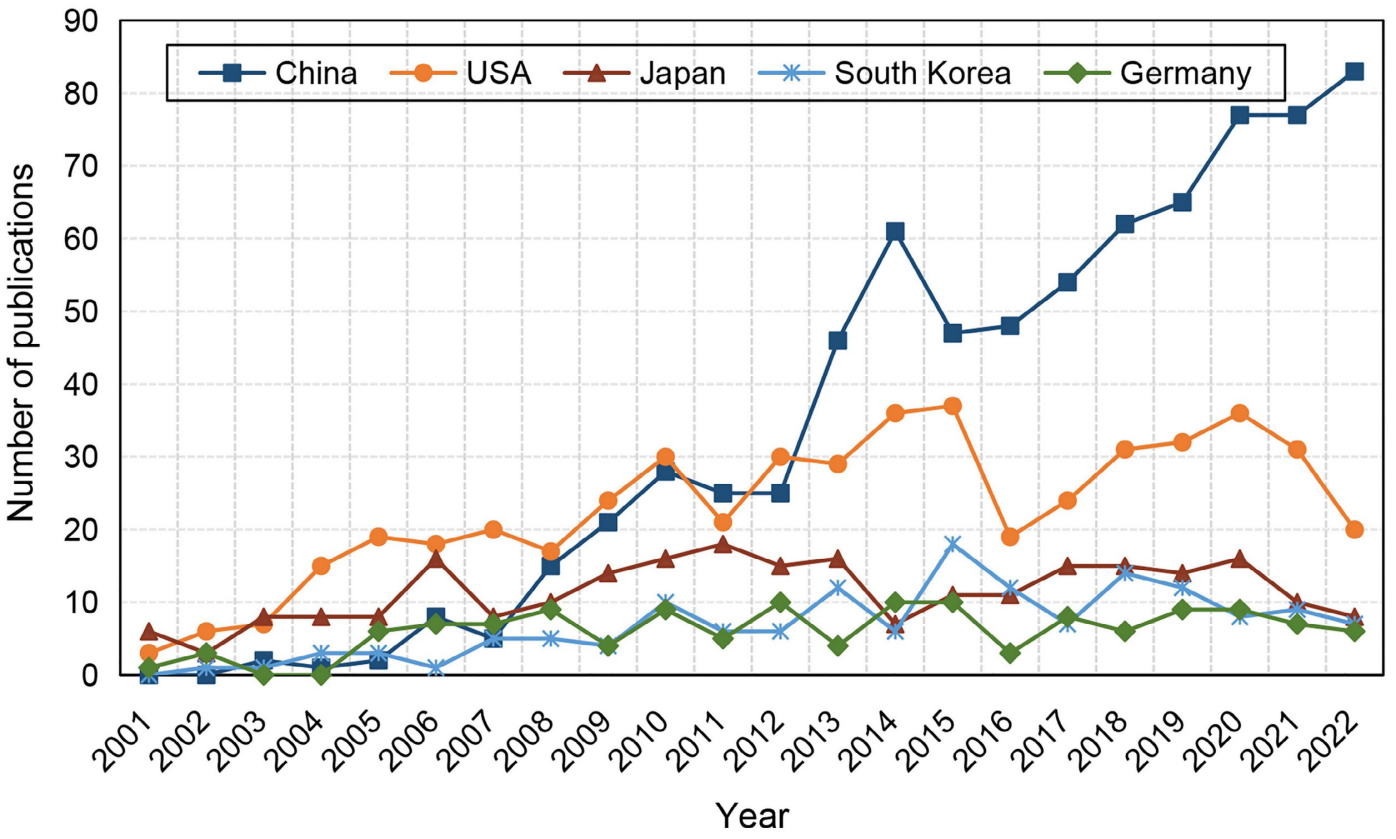
Top 5 countries distribution trends of publications in the field of stem cells and ischemic stroke.

**Figure 4C:**
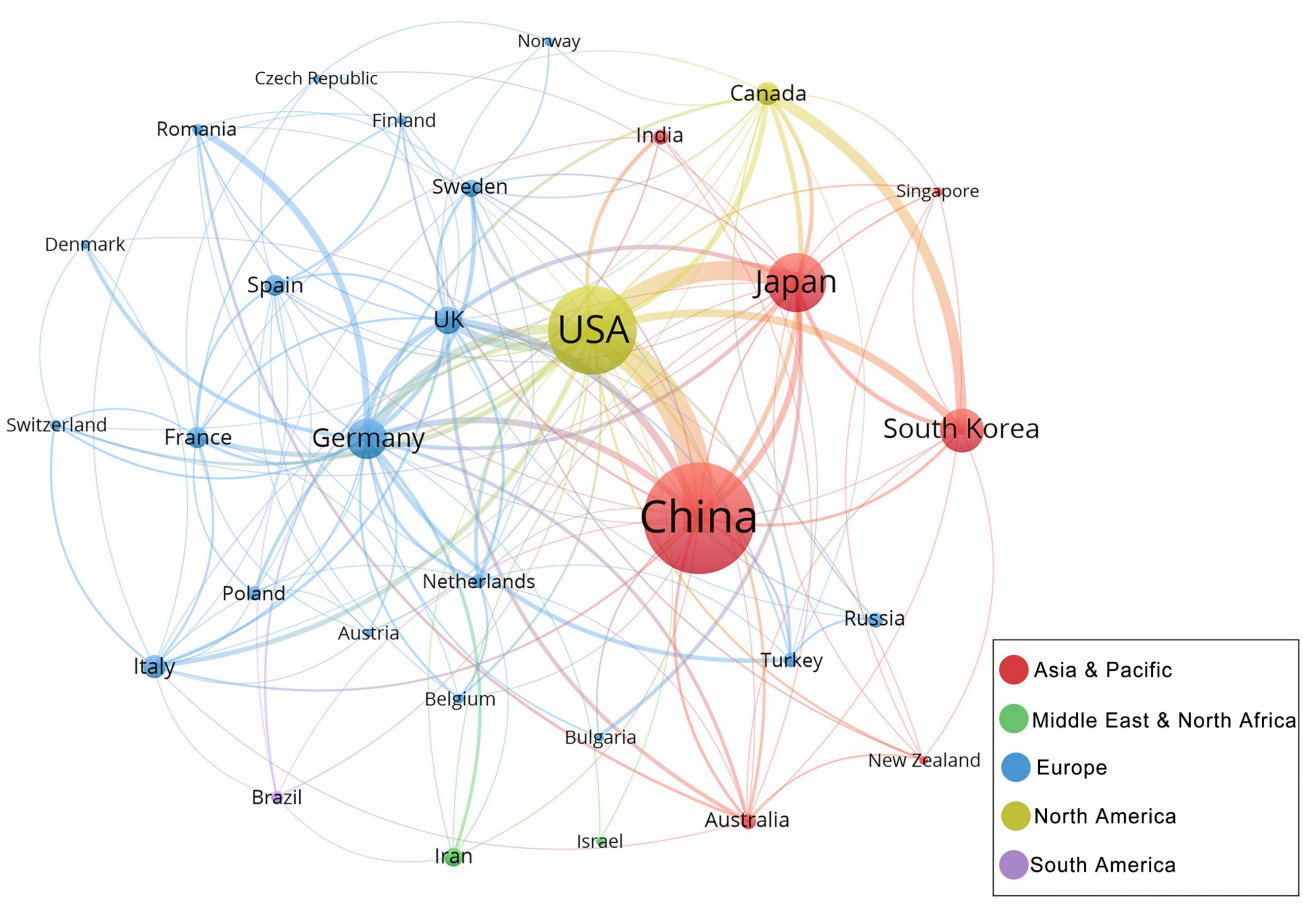
Collaboration networks of the countries with at least 5 publications. Nodes in the network denote countries, and the size of nodes hinges on the number of publications of countries; The curves represent the linkages between different countries, and the size depends on the intensity of cooperation; The colours represent regional distribution.

**Table 1:** Top 10 most productive countries in the field of stem cells and ischemic stroke.

Rank	Country	TP	Percentage (%)	TC	ACPP	H-index
1	China	752	38.92	17581	23.38	59
2	USA	505	26.14	36921	73.11	100
3	Japan	253	13.10	11273	44.56	60
4	South Korea	150	7.76	7648	50.99	42
5	Germany	133	6.88	8020	60.30	44
6	UK	70	3.62	3538	50.54	33
7	Canada	52	2.69	3315	63.75	29
8	Italy	51	2.63	2864	56.16	26
9	Spain	44	2.28	1544	35.09	24
10	France	44	2.28	1360	30.91	22

TP: total publications; TC: total citations; ACPP: average citation per publication.

**Table 2:** Top 10 most productive institutions in the field of stem cells and ischemic stroke.

Rank	Institution	Country	TP	TC	ACPP	H-index
1	University of South Florida	USA	45	1426	31.69	20
2	Henry Ford Hospital	USA	34	4450	130.88	29
3	Oakland University	USA	33	4414	133.76	28
4	Shanghai Jiao Tong University	China	32	1166	36.44	17
5	Yale University	USA	30	1905	63.50	19
6	Shanghai Jiao Tong University Ruijin Hospital	China	29	1500	51.72	20
7	Hyogo College of Medicine	Japan	28	1773	63.32	17
8	Yonsei University	South Korea	27	2604	96.44	17
9	Sapporo Medical University	Japan	26	2601	100.04	20
10	Sungkyunkwan University	South Korea	26	885	34.04	17

TP: total publications; TC: total citations; ACPP: average citation per publication.

**Table 3:** Top 10 most productive journals in the field of stem cells and ischemic stroke.

Rank	Journal	TP	TC	ACPP	H-index	IF (2021)	WOS category (rank/total number of journals in WOS category)
1	Stroke	59	5351	90.69	40	10.17	Clinical Neurology (14/212)
							Peripheral Vascular Disease (7/67)
2	Brain Research	58	2682	46.24	31	3.61	Neurosciences (156/275)
3	Neural Regeneration Research	57	873	15.32	16	6.058	Cell Biology (69/195)
							Neurosciences (61/275)
4	Cell Transplantation	56	1944	34.71	25	4.139	Cell & Tissue Engineering (19/29)
							Medicine, Research & Experimental (75/139)
							Transplantation (9/25)
5	PLoS One	52	2433	46.79	28	3.752	Multidisciplinary Sciences (29/74)
6	Journal of Cerebral Blood Flow and Metabolism	45	3516	78.13	31	6.597	Endocrinology and Metabolism (26/146)
							Hematology (20/78)
							Neurosciences (49/275)
7	Stem Cell Research & Therapy	40	1174	29.35	19	8.088	Cell & Tissue Engineering (4/29)
							Cell Biology (46/195)
							Medicine, Research & Experimental (27/139)
8	International Journal of Molecular Sciences	36	586	16.28	12	6.208	Biochemistry & Molecular Biology (69/297)
							Chemistry, Multidisciplinary (50/179)
9	Neuroscience	33	1903	57.67	21	3.708	Neurosciences (147/275)
10	Experimental Neurology	33	2239	67.85	22	5.62	Neurosciences (72/275)

TP: total publications; TC: total citations; ACPP: average citation per publication; IF: impact factor.

**Table 4:** Top 10 articles with the highest citations in the field of stem cells and ischemic stroke.

Rank	Year	First Author	Title	Journal	Citations
1	2006	Caplan, A.I.	Mesenchymal stem cells as trophic mediators.	Journal of Cellular Biochemistry	2120
2	2004	Imitola, J.	Directed migration of neural stem cells to sites of cns injury by the stromal cell derived factor 1 alpha/cxc chemokine receptor 4 pathway.	Proceedings of the National Academy of Sciences of The United States of America	856
3	2005	Bang, O.Y.	Autologous mesenchymal stem cell transplantation in stroke patients.	Annals of Neurology	845
4	2001	Volpe, J.J.	Neurobiology of periventricular leukomalacia in the premature infant.	Pediatric Research	685
5	2006	Ohab, J.J.	A neurovascular niche for neurogenesis after stroke.	Journal of Neuroscience	668
6	2002	Zhao, L.R.	Human bone marrow stem cells exhibit neural phenotypes and ameliorate neurological deficits after grafting into the ischemic brain of rats.	Experimental Neurology	647
7	2004	Taguchi, A.	Administration of cd34(+) cells after stroke enhances neurogenesis via angiogenesis in a mouse model.	Journal of Clinical Investigation	616
8	2002	Hoehn, M.	Monitoring of implanted stem cell migration *in vivo*: a highly resolved *in vivo* magnetic resonance imaging investigation of experimental stroke in rat.	Proceedings of the National Academy of Sciences of the United States of America	586
9	2010	Lee, J.S.	A long-term follow-up study of intravenous autologous mesenchymal stem cell transplantation in patients with ischemic stroke.	Stem Cells	557
10	2005	Schneider, A.	The hematopoietic factor g csf is a neuronal ligand that counteracts programmed cell death and drives neurogenesis.	Journal of Clinical Investigation	539

**Table 5:** Top 10 co-cited references in the field of stem cells and ischemic stroke.

Rank	Title	Journal	Co-citation	Centrality
1	Neuronal replacement from endogenous precursors in the adult brain after stroke (Arvidsson A, 2002).	Nature Medicine	56	0.19
2	A long-term follow-up study of intravenous autologous mesenchymal stem cell transplantation in patients with ischemic stroke (Lee JS, 2010).	Stem Cells	46	0.68
3	Intravenous administration of auto serum-expanded autologous mesenchymal stem cells in stroke (Honmou O, 2011).	Brain	38	0.56
4	Regeneration of hippocampal pyramidal neurons after ischemic brain injury by recruitment of endogenous neural progenitors (Nakatomi H, 2002).	Cell	36	0.01
5	Neurorestorative therapies for stroke: underlying mechanisms and translation to the clinic (Zhang ZG, 2009).	The Lancet. Neurology	34	0
6	Clinical outcomes of transplanted modified bone marrow-derived mesenchymal stem cells in stroke: A phase 1/2a study (Steinberg GK, 2016).	Stroke	31	0.04
7	Subventricular zone-derived neuroblasts migrate and differentiate into mature neurons in the post-stroke adult striatum (Yamashita T, 2006).	The Journal of Neuroscience	30	0.17
8	Neurogenesis in dentate subgranular zone and rostral subventricular zone after focal cerebral ischemia in the rat (Jin KL, 2001).	Proceedings of the National Academy of Sciences of the United States of America	28	0.03
9	Rat forebrain neurogenesis and striatal neuron replacement after focal stroke (Parent JM, 2002).	Annals of Neurology	28	0.2
10	Stem cells for ischemic brain injury: a critical review (Burns TC, 2009).	Journal of Comparative Neurology	28	0.33

**Table 6:**
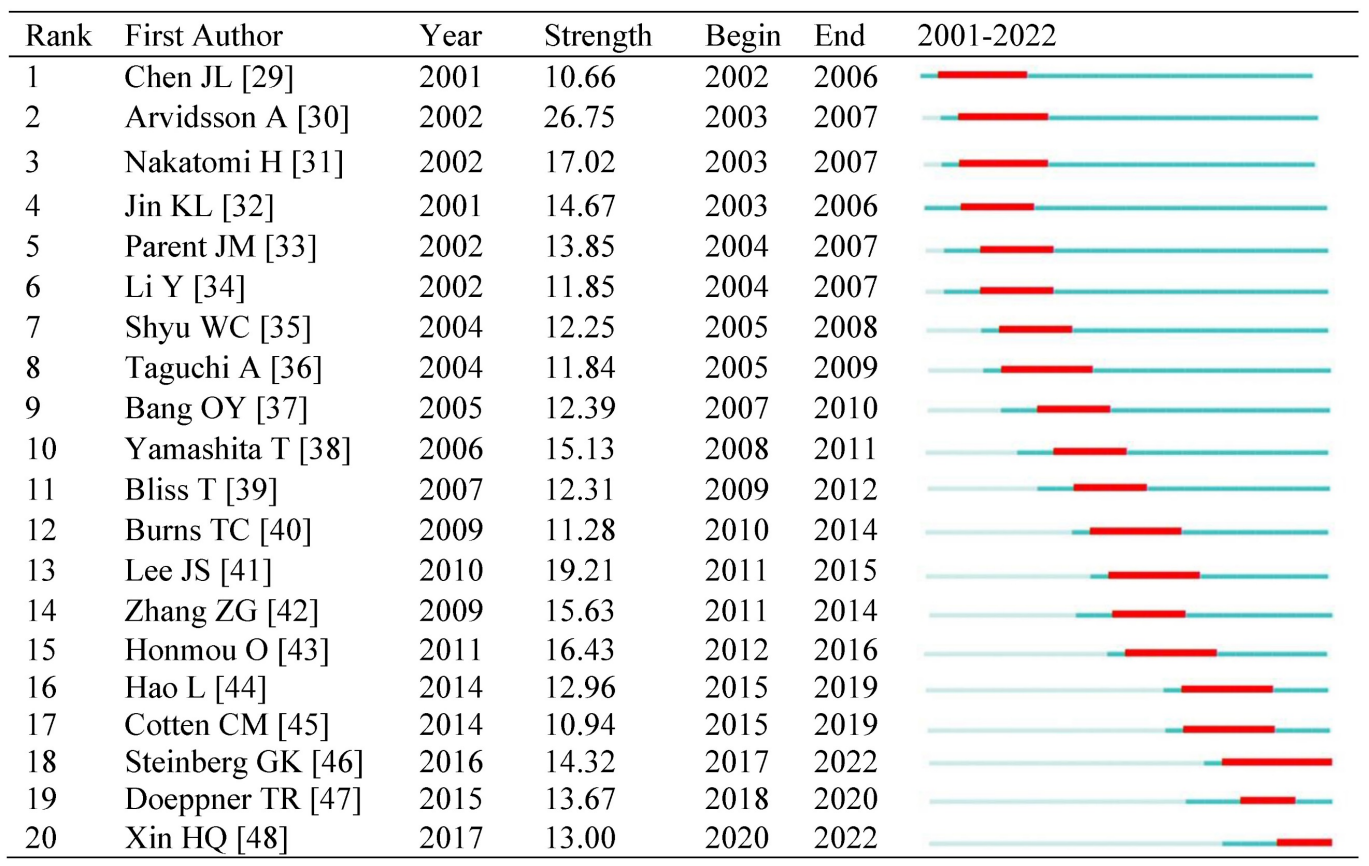
Top 20 references with the strongest citation bursts in stem cells and ischemic stroke research.

## Abbreviations

NPCs: neural precursor cells
SVZ: subventricular zone
MSCs: mesenchymal stem cells
EPCs: endothelial progenitor cells
VPCs: vascular progenitor cells
ESCs: embryonic stem cells
iPSCs: induced pluripotent stem cells
WoS: web of science
SCI-E: science citation index-expanded
SSCI: social sciences citation index
TP: total publications
TC: total citations
ACPP: average citation per publication
SGZ: subgranular zone
NSCs: neural stem cells
OGD: oxygen glucose deprivation
MCAO: middle cerebral artery occlusion
EVs: extracellular vehicles
AAY: average appearing year
VEGF: vascular endothelial growth factor
Ang: angiopoietin
PI3K: phosphatidylinositol 3-kinase
SDF-1α: stromal cell-derived factor 1alpha
CXCR4: CXC chemokine receptor

## References

[1] Lu M, Guo J, Wu B (2021). Mesenchymal stem cell-mediated mitochondrial transfer: a therapeutic approach for ischemic stroke. Transl Stroke Res.

[2] Maida CD, Norrito RL, Daidone M (2020). Neuroinflammatory mechanisms in ischemic stroke: focus on cardioembolic stroke, background, and therapeutic approaches. Int J Mol Sci.

[3] Kim JS (2019). tPA helpers in the treatment of acute ischemic stroke: are they ready for clinical use?. J Stroke.

[4] Li F, Liu WC, Wang Q (2019). NG2-glia cell proliferation and differentiation by glial growth factor 2 (GGF2), a strategy to promote functional recovery after ischemic stroke. Biochem pharmacol 2020; [Epub ahead of print]. DOI: 10.1016/j.bcp.

[5] Wei L, Wei ZZ, Jiang MQ (2017). Stem cell transplantation therapy for multifaceted therapeutic benefits after stroke. Prog Neurobiol.

[6] Zhou L, Zhu H, Bai X (2022). Potential mechanisms and therapeutic targets of mesenchymal stem cell transplantation for ischemic stroke. Stem Cell Res Ther.

[7] Zhang RL, Zhang ZG, Zhang L (2001). Proliferation and differentiation of progenitor cells in the cortex and the subventricular zone in the adult rat after focal cerebral ischemia. Neuroscience.

[8] Hernandez R, Jimenez-Luna C, Perales-Adan J (2020). Differentiation of human mesenchymal stem cells towards neuronal lineage: clinical trials in nervous system disorders. Biomol Ther.

[9] Barzegar M, Wang YP, Eshaq RS (2020). Human placental mesenchymal stem cells improve stroke outcomes via extracellular vesicles-mediated preservation of cerebral blood flow. EBioMedicine 2021; [Epub ahead of print]. DOI: 10.1016/j.ebiom.

[10] Kurozumi K, Nakamura K, Tamiya T (2004). BDNF gene-modified mesenchymal stem cells promote functional recovery and reduce infarct size in the rat middle cerebral artery occlusion model. Mol Ther.

[11] Liu H, Honmou O, Harada K (2006). Neuroprotection by PlGF gene-modified human mesenchymal stem cells after cerebral ischaemia. Brain.

[12] Li J, Tang YH, Wang YT (2014). Neurovascular recovery via cotransplanted neural and vascular progenitors leads to improved functional restoration after ischemic stroke in rats. Stem Cell Rep.

[13] Kolbinger A, Kestner RI, Jencio L (2022). Behind the wall-compartment-specific neovascularisation during post-stroke recovery in mice. Cells.

[14] Sundberg M, Andersson PH, Akesson E (2011). Markers of pluripotency and differentiation in human neural precursor cells derived from embryonic stem cells and CNS tissue. Cell Transplant.

[15] Sarmah D, Kaur H, Saraf J (2018). Getting closer to an effective intervention of ischemic stroke: the big promise of stem cell. Transl Stroke Res.

[16] Lee TK, Lu CY, Tsai ST (2021). Complete restoration of motor function in acute cerebral stroke treated with allogeneic human umbilical cord blood monocytes: preliminary results of a phase I clinical trial. Cell Transplant.

[17] Garcia-Belda P, Prima-Garcia H, Aliena-Valero A (2021). Intravenous SPION-labeled adipocyte-derived stem cells targeted to the brain by magnetic attraction in a rat stroke model: An ultrastructural insight into cell fate within the brain. Nanomedicine 2022; [Epub ahead of print]. DOI: 10.1016/j.nano.

[18] Hur HJ, Lee JY, Kim DH (2022). Conditioned medium of human pluripotent stem cell-derived neural precursor cells exerts neurorestorative effects against ischemic stroke model. Int J Mol Sci.

[19] Ranjbaran M, Vali R, Yaghoobi Z (2022). Adipose-derived mesenchymal stem cells reduced transient cerebral ischemia injury by modulation of inflammatory factors and AMPK signaling. Behav Brain Res 2022; [Epub ahead of print]. DOI: 10.1016/j.bbr.

[20] Hassan W, Yekta BG, Nabavi SM (2023). The progress and research trends of statin medications: advanced epidemiological and bibliometrical assessment. Curr probl cardiol 2023; [Epub ahead of print]. DOI: 10.1016/j.cpcardiol.

[21] Ding Z, Jiang N, Yang T (2022). Mapping the research trends of astrocytes in stroke: A bibliometric analysis. Front Cell Neurosci 2022; [Epub ahead of print]. DOI: 10.3389/fncel.

[22] Zhao J, Yu GY, Cai MX (2018). Bibliometric analysis of global scientific activity on umbilical cord mesenchymal stem cells: a swiftly expanding and shifting focus. Stem Cell Res Ther.

[23] Zhang Q, Zeng Y, Zheng S (2023). Research hotspots and frotiers of stem cells in stroke: A bibliometric analysis from 2004 to 2022. Front Pharmacol 2023; [Epub ahead of print]. DOI: 10.3389/fphar.

[24] Xia DM, Wang XR, Zhou PY (2021). Research progress of heat stroke during 1989-2019: a bibliometric analysis. Mil Med Res.

[25] van Eck NJ, Waltman L (2010). Software survey: VOSviewer, a computer program for bibliometric mapping. Scientometrics.

[26] Chen C (2004). Searching for intellectual turning points: progressive knowledge domain visualization. Proc Natl Acad Sci U S A.

[27] Chen T, Liu YX, Huang L (2022). ImageGP: An easy-to-use data visualization web server for scientific researchers. iMeta.

[28] Leydesdorff L, Comins JA, Sorensen AA (2016). Cited references and Medical Subject Headings (MeSH) as two different knowledge representations: clustering and mappings at the paper level. Scientometrics.

[29] Chen J, Li Y, Wang L (2001). Therapeutic benefit of intravenous administration of bone marrow stromal cells after cerebral ischemia in rats. Stroke.

[30] Arvidsson A, Collin T, Kirik D (2002). Neuronal replacement from endogenous precursors in the adult brain after stroke. Nat Med.

[31] Nakatomi H, Kuriu T, Okabe S (2002). Regeneration of hippocampal pyramidal neurons after ischemic brain injury by recruitment of endogenous neural progenitors. Cell.

[32] Jin K, Minami M, Lan JQ (2001). Neurogenesis in dentate subgranular zone and rostral subventricular zone after focal cerebral ischemia in the rat. Proc Natl Acad Sci U S A.

[33] Parent JM, Vexler ZS, Gong C (2002). Rat forebrain neurogenesis and striatal neuron replacement after focal stroke. Ann Neurol.

[34] Li Y, Chen J, Chen XG (2002). Human marrow stromal cell therapy for stroke in rat: neurotrophins and functional recovery. Neurology.

[35] Shyu WC, Lin SZ, Yang HI (2004). Functional recovery of stroke rats induced by granulocyte colony-stimulating factor-stimulated stem cells. Circulation.

[36] Taguchi A, Soma T, Tanaka H (2004). Administration of CD34+ cells after stroke enhances neurogenesis via angiogenesis in a mouse model. J Clin Invest.

[37] Bang OY, Lee JS, Lee PH (2005). Autologous mesenchymal stem cell transplantation in stroke patients. Ann Neurol.

[38] Yamashita T, Ninomiya M, Hernández Acosta P (2006). Subventricular zone-derived neuroblasts migrate and differentiate into mature neurons in the post-stroke adult striatum. J Neurosci.

[39] Bliss T, Guzman R, Daadi M (2007). Cell transplantation therapy for stroke. Stroke.

[40] Burns TC, Verfaillie CM, Low WC (2009). Stem cells for ischemic brain injury: a critical review. J Comp Neurol.

[41] Lee JS, Hong JM, Moon GJ (2010). A long-term follow-up study of intravenous autologous mesenchymal stem cell transplantation in patients with ischemic stroke. Stem Cells.

[42] Zhang ZG, Chopp M (2009). Neurorestorative therapies for stroke: underlying mechanisms and translation to the clinic. Lancet Neurol.

[43] Honmou O, Houkin K, Matsunaga T (2011). Intravenous administration of auto serum-expanded autologous mesenchymal stem cells in stroke. Brain.

[44] Hao L, Zou Z, Tian H (2014). Stem cell-based therapies for ischemic stroke. Biomed Res Int.

[45] Cotten CM, Murtha AP, Goldberg RN (2014). Feasibility of autologous cord blood cells for infants with hypoxic-ischemic encephalopathy. J Pediatr.

[46] Steinberg GK, Kondziolka D, Wechsler LR (2016). Clinical outcomes of transplanted modified bone marrow-derived mesenchymal stem cells in stroke: a phase 1/2a study. Stroke.

[47] Doeppner TR, Herz J, Görgens A (2015). Extracellular vesicles improve post-stroke neuroregeneration and prevent postischemic immunosuppression. Stem Cells Transl Med.

[48] Xin H, Katakowski M, Wang F (2017). MicroRNA cluster miR-17-92 cluster in exosomes enhance neuroplasticity and functional recovery after stroke in rats. Stroke.

[49] Ogawa M (1993). Differentiation and proliferation of hematopoietic stem cells. Blood.

[50] Mezey E, Chandross KJ, Harta G (2000). Turning blood into brain: cells bearing neuronal antigens generated *in vivo* from bone marrow. Science.

[51] Cui LL, Nitzsche F, Pryazhnikov E (2017). Integrin α4 overexpression on rat mesenchymal stem cells enhances transmigration and reduces cerebral embolism after intracarotid injection. Stroke.

[52] Bai YY, Peng XG, Wang LS (2015). Bone marrow endothelial progenitor cell transplantation after ischemic stroke: an investigation into its possible mechanism. CNS Neurosci Ther.

[53] Liu X, Jia X Neuroprotection of stem cells against ischemic brain injury: from bench to clinic. Transl Stroke Res; in press. DOI: 10.1007/s12975-023-01163-3.

[54] Yang Y, Hu X, Qin Q (2022). Optimal therapeutic conditions for the neural stem cell-based management of ischemic stroke: a systematic review and network meta-analysis based on animal studies. BMC Neurol.

[55] Wu KJ, Yu S, Lee JY (2017). Improving neurorepair in stroke brain through endogenous neurogenesis-enhancing drugs. Cell Transplant.

[56] Jiang XC, Xiang JJ, Wu HH (2019). Neural stem cells transfected with reactive oxygen species-responsive polyplexes for effective treatment of ischemic stroke. Adv Mater.

[57] Gelmi A, Schutt CE (2021). Stimuli-responsive biomaterials: scaffolds for stem cell control. Adv Healthc Mater.

[58] Boese AC, Le QSE, Pham D (2018). Neural stem cell therapy for subacute and chronic ischemic stroke. Stem Cell Res Ther.

[59] Galiakberova AA, Dashinimaev EB (2020). Neural stem cells and methods for their generation from induced pluripotent stem cells *in vitro*. Front Cell Dev Biol.

[60] Zhang GL, Zhu ZH, Wang YZ (2019). Neural stem cell transplantation therapy for brain ischemic stroke: Review and perspectives. World J Stem Cells.

[61] Zhao T, Zhu T, Xie L (2022). Neural stem cells therapy for ischemic stroke: progress and challenges. Transl Stroke Res.

[62] Korshunova I, Rhein S, Garcia-Gonzalez D (2020). Genetic modification increases the survival and the neuroregenerative properties of transplanted neural stem cells. JCI Insight.

[63] Ejma M, Madetko N, Brzecka A (2022). The role of stem cells in the therapy of stroke. Curr Neuropharmacol.

[64] Imitola J, Raddassi K, Park KI (2004). Directed migration of neural stem cells to sites of CNS injury by the stromal cell-derived factor 1 alpha/CXC chemokine receptor 4 pathway. Proc Natl Acad Sci U S A.

[65] Lee JY, Castelli V, Kumar N (2022). Contraceptive drug, Nestorone, enhances stem cell-mediated remodeling of the stroke brain by dampening inflammation and rescuing mitochondria. Free Radic Biol Med.

[66] Cunningham CJ, Redondo-Castro E, Allan SM (2018). The therapeutic potential of the mesenchymal stem cell secretome in ischaemic stroke. J Cereb Blood flow Metab.

[67] Wislet-Gendebien S, Hans G, Leprince P (2005). Plasticity of cultured mesenchymal stem cells: switch from nestin-positive to excitable neuron-like phenotype. Stem Cells.

[68] Pittenger MF, Mackay AM, Beck SC (1999). Multilineage potential of adult human mesenchymal stem cells. Science.

[69] Tian H, Yang X, Zhao J (2023). Hypoxia-preconditioned bone marrow mesenchymal stem cells improved cerebral collateral circulation and stroke outcome in mice. Arterioscler Thromb Vasc Biol.

[70] Liao Y, Ming J, Song W Mitochondrial transplantation and immune response of human bone marrow mesenchymal stem cells for the Therapeutic of ischemic stroke. Curr Stem Cell Res Ther; in press. DOI: 10.2174/1574888X18666230505103407.

[71] Hao L, Yang Y, Xu X (2022). Modulatory effects of mesenchymal stem cells on microglia in ischemic stroke. Front Neurol 2022; [Epub ahead of print]. DOI: 10.3389/fneur.

[72] Jaillard A, Hommel M, Moisan A (2020). Autologous mesenchymal stem cells improve motor recovery in subacute ischemic stroke: a randomized clinical trial. Transl Stroke Res.

[73] Law ZK, Tan HJ, Chin SP (2021). The effects of intravenous infusion of autologous mesenchymal stromal cells in patients with subacute middle cerebral artery infarct: a phase 2 randomized controlled trial on safety, tolerability and efficacy. Cytotherapy.

[74] Asgari Taei A, Khodabakhsh P, Nasoohi S (2022). Paracrine Effects of Mesenchymal Stem Cells in Ischemic Stroke: Opportunities and Challenges. Mol Neurobiol.

[75] Maumus M, Rozier P, Boulestreau J (2020). Mesenchymal stem cell-derived extracellular vesicles: opportunities and Challenges for clinical translation. Front Bioeng Biotechnol.

[76] Cui GH, Wu J, Mou FF (2018). Exosomes derived from hypoxia-preconditioned mesenchymal stromal cells ameliorate cognitive decline by rescuing synaptic dysfunction and regulating inflammatory responses in APP/PS1 mice. FASEB J.

[77] Mennan C, Garcia J, Roberts S (2019). A comprehensive characterisation of large-scale expanded human bone marrow and umbilical cord mesenchymal stem cells. Stem Cell Res Ther.

[78] Vymetalova L, Kucirkova T, Knopfova L (2020). Large-scale automated Hollow-Fiber bioreactor expansion of umbilical cord-derived human mesenchymal stromal cells for neurological disorders. Neurochem Res.

[79] Ohab JJ, Fleming S, Blesch A (2006). A Neurovascular Niche for Neurogenesis after Stroke. J Neurosci.

[80] Yang HT, Chen JC (2022). Bone marrow mesenchymal stem cell-derived exosomes carrying long noncoding RNA ZFAS1 alleviate oxidative stress and inflammation in ischemic stroke by inhibiting microRNA-15a-5p. Metab Brain Dis.

[81] Ye YC, Chang ZH, Wang P (2022). Infarct-preconditioning exosomes of umbilical cord mesenchymal stem cells promoted vascular remodeling and neurological recovery after stroke in rats. Stem Cell Res Ther.

[82] Teixeira FG, Panchalingam KM, Assunção-Silva R (2016). Modulation of the mesenchymal stem cell secretome using computer-controlled bioreactors: impact on neuronal cell proliferation, survival and differentiation. Sci Rep.

