# Supplementary material for: Bibliometric Analysis of Stem Cells in Ischemic Stroke (2001-2022): Trends, Hotspots and Prospects
Source: Int J Med Sci. 2024 Jan 1;21(1):151–68. doi: 10.7150/ijms.86591 (PMC10750336; doi:10.7150/ijms.86591)
Supplement: Supplementary file 1 — Supplementary tables. [file ijmsv21p0151s1.pdf]

Supplementary Material

Table S1 References of figure 1

Rank	Reference
1	Treatment of stroke in rat with intracarotid administration of marrow stromal cells. (Li et al., 2001)
2	Human bone marrow stem cells exhibit neural phenotypes and ameliorate neurological deficits after grafting into the ischemic brain of rats. (Zhao et al., 2002)
3	Bone marrow-derived endothelial progenitor cells participate in cerebral neovascularization after focal cerebral ischemia in the adult mouse. (Zhang et al., 2002)
4	Monitoring of implanted stem cell migration in vivo: a highly resolved in vivo magnetic resonance imaging investigation of experimental stroke in rat. (Hoehn et al., 2002)
5	Stem cell factor stimulates neurogenesis in vitro and in vivo. (Jin et al., 2002)
6	EGF amplifies the replacement of parvalbumin-expressing striatal interneurons after ischemia. (Teramoto et al., 2003)
7	Directed migration of neural stem cells to sites of CNS injury by the stromal cell-derived factor 1alpha/CXC chemokine receptor 4 pathway. (Imitola et al., 2004)
8	Administration of CD34+ cells after stroke enhances neurogenesis via angiogenesis in a mouse model. (Taguchi et al., 2004)
9	Functional recovery of stroke rats induced by granulocyte colony-stimulating factor-stimulated stem cells. (Shyu et al., 2004)
10	The hematopoietic factor G-CSF is a neuronal ligand that counteracts programmed cell death and drives neurogenesis. (Schneider et al., 2005)
11	Autologous mesenchymal stem cell transplantation in stroke patients. (De Keyser J, 2005)
12	Comparison of ischemia-directed migration of neural precursor cells after intrastriatal, intraventricular, or intravenous transplantation in the rat. (Jin et al., 2005)
13	Transplantation of embryonic stem cells overexpressing Bcl-2 promotes functional recovery after transient cerebral ischemia. (Wei et al., 2005)
14	BDNF gene-modified mesenchymal stem cells promote functional recovery and reduce infarct size in the rat middle cerebral artery occlusion model. (Kurozumi et al., 2004)
15	Neuroprotection by PlGF gene-modified human mesenchymal stem cells after cerebral ischaemia. (Liu et al., 2006)
16	Intracarotid transplantation of bone marrow stromal cells increases axon-myelin remodeling after stroke. (Shen et al., 2006)

17	Delayed post-ischaemic neuroprotection following systemic neural stem cell transplantation involves multiple mechanisms. (Bacigaluppi et al., 2009)
18	Functional improvement of focal cerebral ischemia injury by subdural transplantation of induced pluripotent stem cells with fibrin glue. (Chen et al., 2010)
19	Adipose-derived mesenchymal stem cells markedly attenuate brain infarct size and improve neurological function in rats. (Leu et al., 2010)
20	A long-term follow-up study of intravenous autologous mesenchymal stem cell transplantation in patients with ischemic stroke. (Lee et al., 2010)
21	Effects of intravenous administration of allogenic bone marrow- and adipose tissue-derived mesenchymal stem cells on functional recovery and brain repair markers in experimental ischemic stroke. (Gutiérrez-Fernández et al., 2013)
22	Extracellular vesicles improve post-stroke neuroregeneration and prevent postischemic immunosuppression. (Doeppner et al., 2015)
23	Human neural stem cells in patients with chronic ischaemic stroke (PISCES): a phase 1, first-in-man study. (Kalladka et al., 2016)
24	MicroRNA cluster miR-17-92 cluster in exosomes enhance neuroplasticity and functional recovery after stroke in rats. (Xin et al., 2017)
25	The therapeutic potential of the mesenchymal stem cell secretome in ischaemic stroke. (Cunningham et al., 2018)
26	Neuroinflammation as a target for treatment of stroke using mesenchymal stem cells and extracellular vesicles. (Dabrowska et al., 2019)
27	Adipose-derived mesenchymal stem cells reduce autophagy in stroke mice by extracellular vesicle transfer of miR-25. (Kuang et al., 2020)
28	Small extracellular vesicles secreted by human iPSC-derived MSC enhance angiogenesis through inhibiting STAT3-dependent autophagy in ischemic stroke. (Xia et al., 2020)

(The literature of the same year was listed from top to bottom and from left to right.)

Table S2 The search terms of this study

Search term	
#1	(TI=((("anterior choroidal artery" OR brain OR cerebellar OR cerebral OR cortical OR hemisphere* OR encephalopathy OR insular OR "internal carotid artery" OR medullary OR midbrain OR "occipital lobe" OR "parietal lobe" OR pontine OR subcortical OR "temporal lobe" OR "vertebrobasilar artery") AND infarct*) OR "isch*emia stroke*" OR "brain isch*emia" OR "brain stem isch*emia" OR "cerebral isch*emia" OR "cerebral stem isch*emia" OR "isch*emic encephalopathy")) AND TI=("stem cell*" OR "mesenchymal stromal cell*" OR "progenitor cell*" OR "precursor cell*" OR "colony forming unit" OR hemangioblast* OR "regenerative cells" OR "embryon* cell*" OR "lipoaspirate cell*")
#2	(AK=((("anterior choroidal artery" OR brain OR cerebellar OR cerebral OR cortical OR hemisphere* OR encephalopathy OR insular OR "internal carotid artery" OR medullary OR midbrain OR "occipital lobe" OR "parietal lobe" OR pontine OR subcortical OR "temporal lobe" OR "vertebrobasilar artery") AND infarct*) OR "isch*emia stroke*" OR "brain isch*emia" OR "brain stem isch*emia" OR "cerebral isch*emia" OR "cerebral stem isch*emia" OR "isch*emic encephalopathy")) AND AK=("stem cell*" OR "mesenchymal stromal cell*" OR "progenitor cell*" OR "precursor cell*" OR "colony forming unit" OR hemangioblast* OR "regenerative cells" OR "embryon* cell*" OR "lipoaspirate cell*")
#3	(AB=((("anterior choroidal artery" OR brain OR cerebellar OR cerebral OR cortical OR hemisphere* OR encephalopathy OR insular OR "internal carotid artery" OR medullary OR midbrain OR "occipital lobe" OR "parietal lobe" OR pontine OR subcortical OR "temporal lobe" OR "vertebrobasilar artery") AND infarct*) OR "isch*emia stroke*" OR "brain isch*emia" OR "brain stem isch*emia" OR "cerebral isch*emia" OR "cerebral stem isch*emia" OR "isch*emic encephalopathy")) AND AB=("stem cell*" OR "mesenchymal stromal cell*" OR "progenitor cell*" OR "precursor cell*" OR "colony forming unit" OR hemangioblast* OR "regenerative cells" OR "embryon* cell*" OR "lipoaspirate cell*")
#4	PY = "2001-2022"
#5	Article type = "Article" and "Review"
#6	Language = "English"
#7	(#1 OR #2 OR #3) AND #4 AND #5 AND #6

Table S3 The primary content of top 20 references with strongest citation bursts

Rank	Year	Primary Content of Research	Strength
1	2001	Therapeutic benefit of intravenous administration of bone marrow stromal cells after ischemic stroke	10.66
2	2002	Neuronal replacement from endogenous neural precursors induced by ischemic stroke	26.75
3	2002	Regenerated hippocampal neurons from endogenous progenitors ameliorating neurological deficits after ischemic stroke	17.02
4	2001	Neurogenesis in dentate subgranular zone and rostral subventricular zone after ischemic stroke	14.67
5	2002	Neurogenesis in subventricular zone and neuroblast migration to lesions after ischemic stroke	13.85
6	2002	Human marrow stromal cell derived neurotrophins in functional recovery after ischemic stroke	11.85
7	2004	Granulocyte colony-stimulating factor-stimulated stem cells for functional recovery of ischemic stroke	12.25
8	2004	Systemic administration of human cord blood-derived CD34(+) cells enhancing neurogenesis via angiogenesis after ischemic stroke	11.84
9	2005	Safety and effeciency of autologous mesenchymal stem cell transplantation in ischemic stroke patients	12.39
10	2006	Subventricular zone-derived neuroblasts in neuronal regeneration following ischemic stroke	15.13
11	2007	A review on the potential mechanisms of transplanted stem cells and critical parameters for transplantation success in ischemic stroke	12.31
12	2009	The potential application of endogenous and exogenous stem cell therapies for ischemic stroke	11.28
13	2010	A long-term follow-up study of mesenchymal stem cell transplantation in patients with ischemic stroke	19.21
14	2009	Mechanisms and translation to the clinic of stem cell-based therapy for ischemic stroke	15.63
15	2011	Feasibility and safety of auto serum-expanded autologous mesenchymal stem cells in ischemic stroke patients	16.43
16	2014	Review of the application progress and current concerns regarding several stem cells employed in ischemic stroke	12.96
17	2014	Feasibility of autologous cord blood cells for infants with hypoxic-ischemic encephalopathy	10.94
18	2016	Safety and effect of modified bone marrow-derived mesenchymal stem cells transplantation to chronic ischemic stroke patients	14.32
19	2015	Mesenchymal stem cell-derived extracellular vesicles improving post-stroke neuroregeneration	13.67
20	2017	Exosomes enriched with microRNA cluster enhancing neural plasticity and functional recovery after ischemic stroke	13.00

Table S4 Top 30 most frequent keywords in the field of ischemic stroke and stem cells

Rank	Keyword	Frequency
1	Cerebral ischemia	608
2	Stroke	452
3	Cerebral ischemic stroke	352
4	Neural stem cells	291
5	Mesenchymal stem cells	263
6	Neurogenesis	260
7	Ischemia	228
8	Stem cells	222
9	Transplantation	175
10	Angiogenesis	169
11	MCAO	158
12	Neuroprotection	153
13	Therapy	145
14	Rats	145
15	Neural progenitor cells	125
16	Bone marrow mesenchymal stem cells	124
17	Cell proliferation	121
18	Cell therapy	110
19	Apoptosis	104
20	Inflammation	100
21	Reperfusion injury	98
22	Mice	92
23	Cell differentiation	92
24	Endothelial progenitor cells	91
25	Nerve regeneration	83
26	Brain injury	82
27	Mirna	82
28	MRI	80
29	Neurons	76
30	Hypoxic ischemic encephalopathy	74

MCAO: middle cerebral artery occlusion; MRI: magnetic resonance imaging.
